# Global Patterns and Drivers of Avian Extinctions at the Species and Subspecies Level

**DOI:** 10.1371/journal.pone.0047080

**Published:** 2012-10-08

**Authors:** Judit K. Szabo, Nyil Khwaja, Stephen T. Garnett, Stuart H. M. Butchart

**Affiliations:** 1 Research Institute for the Environment and Livelihoods, Charles Darwin University, Darwin, Northern Territory, Australia; 2 BirdLife International, Cambridge, United Kingdom; University of Durham, United Kingdom

## Abstract

Birds have long fascinated scientists and travellers, so their distribution and abundance through time have been better documented than those of other organisms. Many bird species are known to have gone extinct, but information on subspecies extinctions has never been synthesised comprehensively. We reviewed the timing, spatial patterns, trends and causes of avian extinctions on a global scale, identifying 279 ultrataxa (141 monotypic species and 138 subspecies of polytypic species) that have gone extinct since 1500. Species extinctions peaked in the early 20^th^ century, then fell until the mid 20^th^ century, and have subsequently accelerated. However, extinctions of ultrataxa peaked in the second half of the 20^th^ century. This trend reflects a consistent decline in the rate of extinctions on islands since the beginning of the 20^th^ century, but an acceleration in the extinction rate on continents. Most losses (78.7% of species and 63.0% of subspecies) occurred on oceanic islands. Geographic foci of extinctions include the Hawaiian Islands (36 taxa), mainland Australia and islands (29 taxa), the Mascarene Islands (27 taxa), New Zealand (22 taxa) and French Polynesia (19 taxa). The major proximate drivers of extinction for both species and subspecies are invasive alien species (58.2% and 50.7% of species and subspecies, respectively), hunting (52.4% and 18.8%) and agriculture, including non-timber crops and livestock farming (14.9% and 31.9%). In general, the distribution and drivers of subspecific extinctions are similar to those for species extinctions. However, our finding that, when subspecies are considered, the extinction rate has accelerated in recent decades is both novel and alarming.

## Introduction

The study of extinction is fundamental to conservation, and understanding trends in the taxonomic and geographic patterns and drivers of extinction may improve our chances of minimising the rate of future human-induced extinctions. Current and recent rates of extinction are unprecedented in human history [1], and may be occurring at rates 2–3 orders of magnitude above the background [2]. The extinction rate is particularly well documented for birds, because of the fascination they held for early scientists and travellers. Since 1500, 150 bird species may have been lost globally [3]. Among them, 132 have been classified as ‘Extinct’ and four as ‘Extinct in the Wild’ (with populations only surviving in captivity). Recognising that it is difficult to determine if the last individual of a population has died, and hence that documenting extinctions is challenging, a further 14 species have been classified as ‘Critically Endangered (Possibly Extinct)’ and one as ‘Critically Endangered (Possibly Extinct in the Wild)’ [4].

Although the reason for extinction can rarely be pinned to a single cause, extinction most often occurs when new threats develop that are outside the evolutionary experience of species [5]. Naive island birds that have never encountered humans or their animal companions have been particularly susceptible. Since human sea-farers began visiting remote islands, extinction has followed in their footsteps [6]–[8]. The fragility of island ecosystems is well known, and along with plants, snails [9] and reptiles [10], birds are among the hardest hit. Around 90% of bird species extinctions have happened on islands [11], [12], although extinction hotspots have changed through time, as sensitive species have been wiped out, leaving only more resilient species, and as new threats have emerged.

The Convention on Biological Diversity aims to conserve biodiversity across all levels, from genes to populations, species and ecosystems [13]. Little information is available on loss of genetic diversity (except in cultivated crops and domestic animal breeds [14]), while loss of biodiversity at the population level is largely restricted to indicators of mean abundance [15], [16], with few metrics relating to loss of subspecific diversity. For example, while avian extinctions have been previously reviewed at the species level [4], [17]–[20], to our knowledge, there has been no analysis of the extent and pattern of loss among subspecies. While some authors oppose the use of subspecies as conservation units given the level of genetic distinction is usually lower than between species [21], others endorse their value [22] and in some countries, such as the US [23] and Australia [24], threatened subspecies of non-threatened species are listed in legislation and receive conservation funding. Also, while there are analyses of losses at the population level for some taxa [25], subspecies are the finest level of genetic variability for which there is knowledge at a global scale for an entire class of animals.

Here we provide the most up-to-date list of extinctions of birds at both species and subspecies levels (see Supplementary Online Material for full lists), analysing the timing, distribution and drivers of global extinctions since 1500 at the finest taxonomic resolution possible (i.e. among the world's 28,183 ultrataxa, comprising 6,440 monotypic species and 21,743 subspecies of polytypic species according to Dickinson [26]). We also extend the analysis of Butchart et al. [4] to identify those ‘Possibly Extinct’ subspecies that are likely to have gone extinct, but the loss of which is unconfirmed.

## Materials and Methods

Data on taxa extinct at the species level were taken from BirdLife International [3] and references therein, and combined with data on subspecies extinctions compiled from a number of sources, including Dickinson [26], del Hoyo et al. (1990–2011), regional and national field guides, family monographs and the scientific literature (Table S1). A preliminary list was reviewed by regional and national experts, and further input was solicited through discussion forums and email list-servers. The former distribution, year of last record, timing and intensity of unsuccessful searches, and apparent drivers of extinction were documented in each case. Following the approach of Butchart et al. [4] we classed potentially extinct taxa as 1) Extinct (where there is no reasonable doubt that the last individual has died), 2) Critically Endangered (Possibly Extinct), hereafter abbreviated to ‘Possibly Extinct’, which are likely, on the balance of evidence, to be extinct, but for which there is a small chance that they may be extant and thus should not be listed as extinct until adequate surveys have failed to find the taxa and unconfirmed reports have been discounted) or 3) extant (likely to survive). This classification required assessing each taxon's predisposition to extinction (e.g., flightlessness, naturally highly restricted range), difficulty of detection (e.g., cryptic colouration, nocturnal or skulking habits, shyness), survey effort (intensity, extensiveness, timing and techniques), properties of the remaining habitat (quantity, quality and suitability), intensity of threats, and the timing and certainty of records. Through evaluating these factors, a judgement was made about whether the lack of recent records was more likely to be because of inadequacy of searches or difficulty of detection, or because of extinction having occurred owing to intense threats (see [4] and [27] for further details).

In this study, most analyses were performed at the finest taxonomic scale by pooling extinct monotypic species with extinct subspecies of polytypic species (referred to as ‘ultrataxa’ following Schodde and Mason [28], with subspecies and full species examined separately in some analyses to highlight differences. For those species with two or more subspecies all of which are extinct, we treated the multiple subspecies as independent units in the calculations. Species-level taxonomy followed BirdLife International [29], and subspecies-level taxonomy followed Dickinson [26] (i.e. only taxa recognised by this source were included as subspecies) with six exceptions: Tuamotu Ground-dove *Gallicolumba erythroptera pectoralis*, Antioquia Brown-banded Antpitta *Grallaria milleri gilesi*, Namoi Grasswren *Amytornis textilis inexpectatus*, Large-tailed Grasswren *A. t. macrourus*, Raiatea Reed-warbler *Acrocephalus caffer musae* and Lord Howe Pigeon *Columba vitiensis godmanae* (see Table S1 for justifications). Taxa considered extinct and whose validity is recognised by other authors but not by BirdLife International [29] or Dickinson [26] are listed in Table S2. We analysed the taxonomy of recent extinctions at the family level (taking the number of extant species and subspecies per family from Dickinson [26]) and calculated for each family the proportion of (a) extinct ultrataxa (279 in total out of 26,073 across all families), (b) extinct species (141 out of 10,049), and (c) threatened species (1,253 out of 9,853 non-Data Deficient species). To assess whether these proportions are significantly larger or smaller than expected by chance, some previous studies [30], [31] have used the binomial equation to calculate the probability of obtaining a value equal to, or larger than, the observed value for each family. However, following the recommendations of Lockwood et al. [32] we used a Monte Carlo simulation approach, removing ultrataxa from families randomly until the observed number of extinctions was reached, ensuring that the number of ultrataxa removed from a family did not exceed the total number of members. From the simulated distribution we calculated the probability of the observed number of extinctions occurring if the null hypothesis was true for each family. We assessed significance using a two-tailed distribution. The lower *p* value was calculated from the number of simulations that resulted in extinction totals at or below the number of extinctions observed in that family divided by the total number of simulations (50,000). Similarly, the upper *p* value was calculated from the number of simulations that resulted in extinction totals at or exceeding the number of observed extinctions divided by the number of simulations. In such an approach, the potential accumulation of decision errors is high and the adjustment of significance levels and/or *p* values is recommended [33]. Although Bonferroni adjustments have been used by some authors [32], [34], others consider these unnecessarily conservative [35], [36], so we calculated *q* values in addition to *p* values to assess whether a particular family had an unusually high or low number of extinctions [37]. While the *p* value is a measure of significance in terms of the false positive rate (i.e. the rate that truly null features are called significant), the *q* value is a measure in terms of the False Discovery Rate (i.e. the rate that significant features are truly null) in a Bayesian framework [38]. We assessed significance at a 5% level (0.025 at each tail). These calculations were conducted using the software QVALUE via the program R ver. 2.15.0.

An estimated date of extinction was assigned to each taxon. Where previous authors had not specified a date on the basis of known records and searches, we took the midpoint between the date of the last confirmed record of the taxon and the date of first survey that subsequently failed to find it. Where there was no information on subsequent surveys, the date of the last record was used. Recognising the uncertainty over estimated dates of extinction, we analysed trends over time using the extinction rate per quarter-century.

The threats believed to have driven each taxon extinct were coded using the IUCN Red List threats classification scheme, derived from Salafsky et al. [39], and scored as primary (if they are estimated to have driven the majority of the decline to extinction, or secondary (if they were a significant contributory factor, causing 10–49% of the decline to extinction). In some cases, particularly for extinctions that occurred longer ago, the drivers were inferred from known drivers driving declines in sympatric species, or inferred from the chronology of threats and/or susceptibility of taxa. For the analysis of threats, we included both primary and secondary drivers. As threats are not currently recorded for extant subspecies, the comparison of past and current drivers of extinctions was conducted at a species level, using only those threats coded as having a high or medium-impact (see [40] for details).

We defined oceanic islands as landmasses that have never been connected to a continental area by a land-bridge and are volcanic in origin, determining this from a wide variety of sources (e.g. [41]–[43]). Continental islands were defined as smaller landmasses on the continental shelf in waters generally less than 200 m deep. For the analyses presented here, Australia was treated as a continent, but Madagascar, New Zealand and New Caledonia were all treated as oceanic islands.

## Results

In total, 138 subspecies (see below) have gone extinct since 1500, 98 of which are confirmed, plus 39 that are highly likely, but not yet confirmed extinct, and hence qualify for Possibly Extinct status (Table S1). One additional subspecies (Guam Kingfisher *Todiramphus cinnamominus cinnamominus*) is Extinct in the Wild. A number of other taxa considered extinct by other authors, we consider definitely or likely to be extant (Table S3). Of the 138 extinct subspecies, 21 relate to nine species in which all subspecies are extinct (Table 1). Thus in total 227 taxa (full species and subspecies of still extant species) have gone extinct since 1500.

**Table 1:** Number of Extinct and Possibly Extinct taxa.

	Extinct	Extinct in the Wild	Possibly Extinct	Possibly Extinct in the Wild
Monotypic species	123	4	13	1
Subspecies of extant species	77	1	39	0
Subspecies of extinct species	21*	-	-	-

* ( = 9 species).

Recent extinctions have not been random with respect to taxonomy. Among speciose families, Psittacidae (parrots), Rallidae (rails), Fringillidae (finches) and Columbidae (pigeons) have suffered a disproportionately large number of extinctions at both the species and ultrataxon level (Table S4). Taxa belonging to these families comprised 36.9% of all extinct taxa. The Dromaiidae (emus), Raphidae (dodo and solitaires), Acanthisittidae (New Zealand wrens) and Mohoidae (Hawaiian honeyeaters) have all lost more than half of their taxa in the last 500 years, although all are families that contained fewer than eight taxa. At the ultrataxon (but not species) level we found that Acanthisittidae, Anatidae (ducks, geese and swans), Dromaiidae, Scolopacidae (sandpipers), Maluridae (Australasian wrens), Strigidae (owls), Sturnidae (starlings), Turdidae (thrushes) and Oriolidae (orioles and figbirds) have experienced a disproportionately high recent extinction rate, suggesting a vulnerability missed by previous studies. Rallidae, Raphidae, Columbidae, Psittacidae, Mohoidae and Fringillidae are the most extinction-prone families at both the species and ultrataxon level. Less speciose families have experienced disproportionately higher extinction rates (Spearman rank correlation, Rs  = −0.356, p<0.001). The pattern was the same for ultrataxa (Rs  = −0.353, p<0.001).

One family has suffered significantly fewer extinctions than expected by chance at the ultrataxon level: Timaliidae (babblers and parrotbills, 0 extinctions/931 ultrataxa). For other speciose families with no extinctions (e.g. Furnariidae (ovenbirds, 605 ultrataxa), Thamnophilidae (antbirds,556), Accipitridae (hawks and eagles, 550), Nectariniidae (sunbirds, 480), Pycnonotidae (bulbuls, 428) and Alaudidae (larks, 415)) the *p* value was significant, but not the *q* value. Passerines comprise 64.4% of all ultrataxa, but only 43.7% of extinctions.

Among families containing species currently threatened, 10 had a significantly larger number of threatened species than expected, and four of these families have not yet suffered any taxon extinctions.

**Figure 1:**
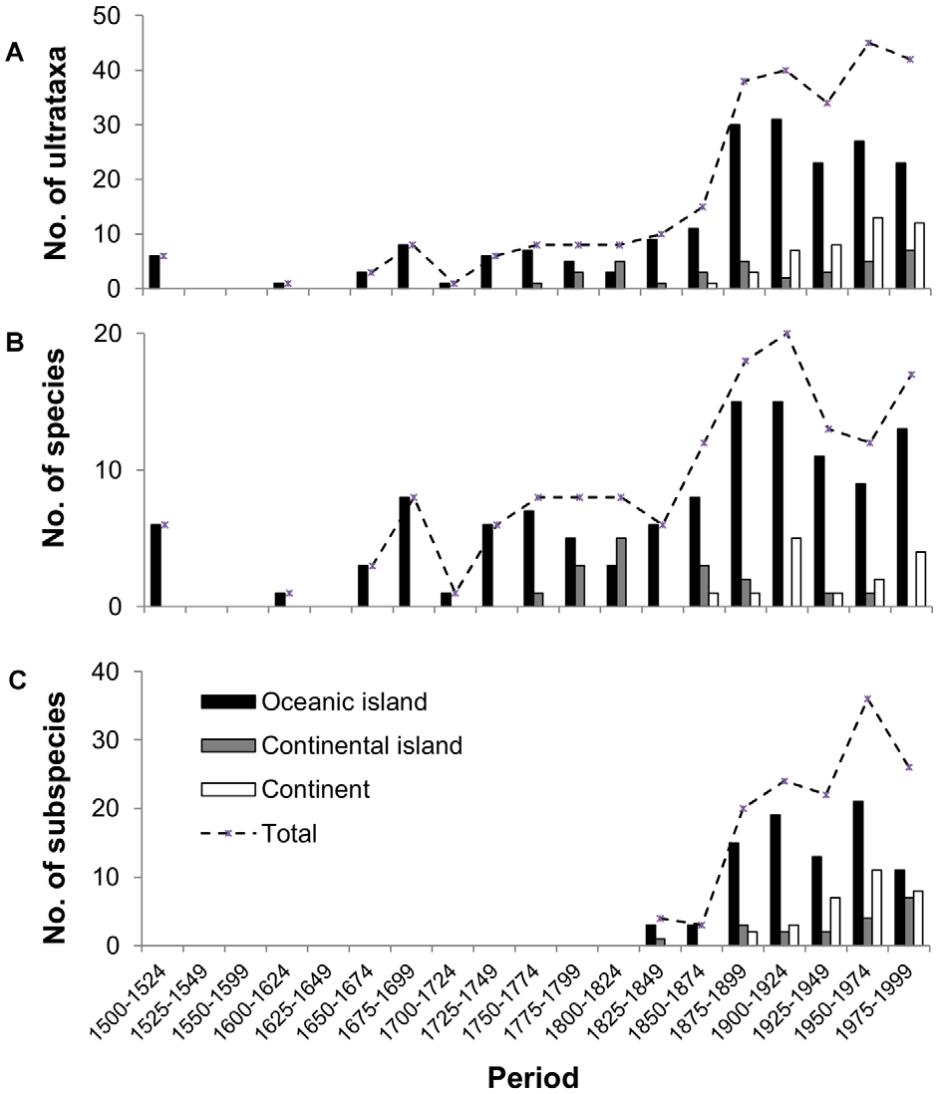
Number of extinctions per 25-year period by landmass (black: oceanic island, grey: continental island, white: continent) for A) ultrataxa (n_ultrataxa_  = 279), B) species (n_sp_  = 150) and C) subspecies (n_ssp_  = 138). Totals include ultrataxa classified as Extinct (n_sp_  = 125 and n_ssp_  = 92), Extinct in the Wild (n_sp_  = 4 and n_ssp_  = 1), Possibly Extinct (n_sp_  = 12 and n_ssp_  = 42) and Possibly Extinct in the Wild (n_sp_  = 1 and n_ssp_  = 0). The dashed line indicates the number of extinctions per 25-year period for all landmass types combined. Since 2000, two subspecies and two species extinctions have been recorded on oceanic islands, none on continental islands and a subspecies and a species on continents.

The overwhelming majority of extinctions have been on oceanic or continental islands, at both the species and subspecies level (78.7% of species extinctions and 63.0% of subspecies extinctions occurring on oceanic islands, compared to 10.6% and 13.8% on continental islands and 9.9% and 23.2% on continents for species and subspecies, respectively). This means that 198 ultrataxa have gone extinct on oceanic islands, 35 on continental islands and 46 on continents. The number of extinctions per 25 years peaked in the last quarter of the 19^th^ century and first quarter of the 20^th^ century for oceanic and continental islands, but appears to have declined subsequently. The first continental extinctions were recorded in the mid-19th century (Great Auk *Pinguinus impennis* in 1852 and Labrador Duck *Camptorhynchus labradorius* in 1875, according to best estimates) and the rate has increased steadily since, with 12 ultrataxon extinctions occurring on continents in the last quarter of the 20^th^ century (Figure 1). Hence, the geographic pattern of extinctions appears to be shifting from oceanic islands to continents. Importantly, this acceleration in the extinction rate on continents has more than compensated for the decline in island extinctions, so the overall rate of extinctions has accelerated since the mid-20^th^ century. Butchart et al. [4] hinted that this phenomenon might be expected, but our analysis at the ultrataxon level is the first to demonstrate it.

**Figure 2:**
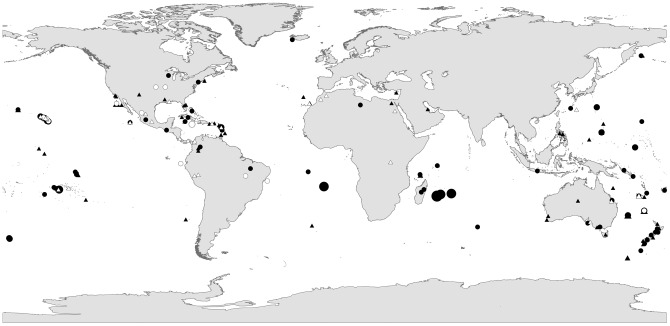
Geographic location of extinctions since 1500. Circles indicate species, triangles indicate subspecies, open symbols indicate ‘Extinct’ taxa, solid symbols indicate ‘Possibly Extinct’ taxa. Larger symbols indicate larger numbers of taxa, as illustrated for species.

**Figure 3:**
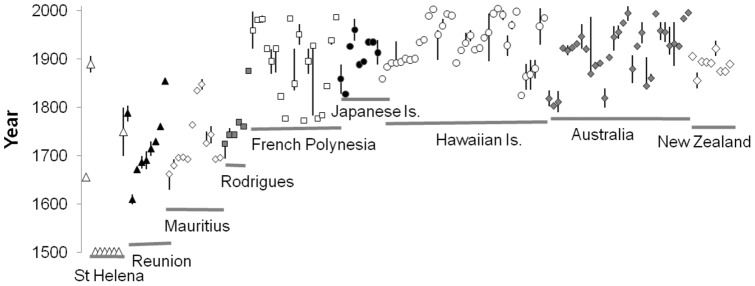
Date of ultrataxa extinctions (with vertical lines showing minimum and maximum estimate of date where available) for selected locations, compared with date of first settlement of >5 **years by a continental or continental-island based nation (grey line).**

**Figure 4:**
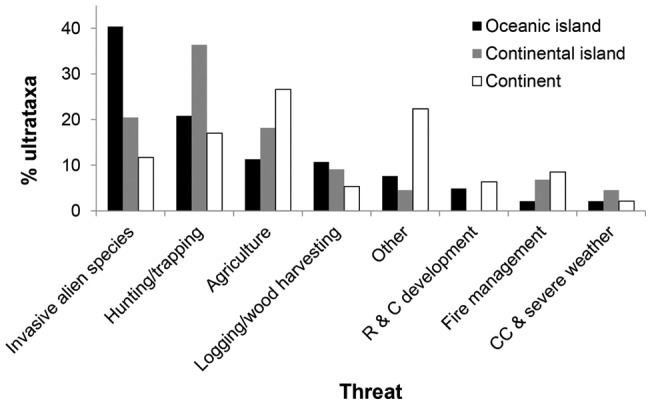
Drivers of extinction (including both primary and secondary threats) on oceanic islands (black), continental islands (grey) and continents (white). “Other” includes: Energy production and mining, Transportation and service corridors, Gathering terrestrial plants, Harvest aquatic resource, Human intrusions and disturbance, Water management/use, Other ecosystem modifications, Introduced genetic material, Pollution and Geological events. Abbreviations: R & C development: Residential and commercial development, CC & severe weather: Climate change and severe weather.

Extinction hotspots include the Caribbean (13 monotypic species +14 subspecies), Hawaiian Islands (23+13), New Zealand (12+10), French Polynesia (11+8), Australia (9+20), and Mexico (5+9; Figure 2). In many cases the timing of extinctions can be related to the year of human colonisation by technologically advanced people (e.g. from mainland Japan to the Japanese Islands; Figure 3), with a substantial number of extinctions typically occurring within a century of colonisation. The impacts of invasive alien species, unsustainable hunting and trapping by humans and unsustainable agriculture have been the major causes of recent avian extinctions (Figure 4). Including both primary and secondary drivers of extinction, invasive alien species have been implicated in the extinction of 82 species and 70 subspecies. Invasive aliens impact native species in different ways: predation (117 ultrataxa), disease (29), habitat degradation (31) and competition (6).

Drivers of extinction have differed among landmass types (Figure 4). The most important have been habitat loss and degradation driven by agriculture on continents, hunting on continental islands and invasive alien species on oceanic islands.

**Figure 5:**
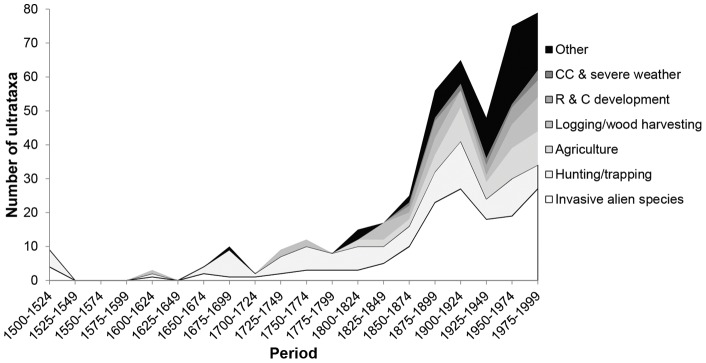
Number of avian ultrataxa extinctions per threat (including both primary and secondary threats for 25-year periods. Abbreviations: R & C development: Residential and commercial development, CC & Severe weather: Climate change and severe weather.

The number of extinctions caused by different threats has changed through time, with climate change/severe weather (principally the latter, but both are combined in the IUCN classification scheme) and residential/commercial development becoming more important over the last century or so (Figure 5).

**Figure 6:**
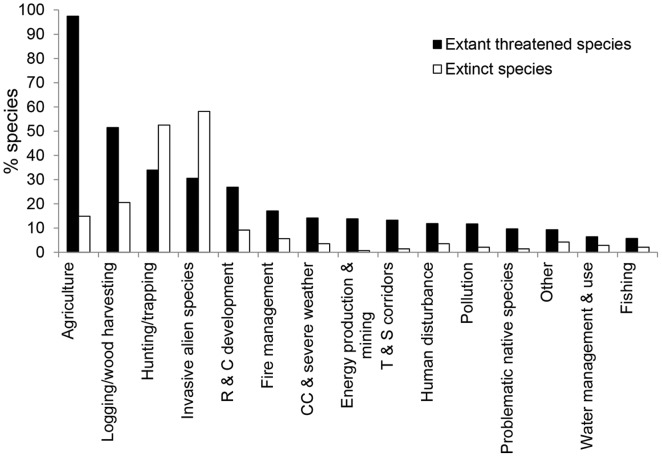
Importance of drivers of species-level extinctions (including both primary and secondary threats) compared with threats to extant species. Abbreviations: R & C development: Residential and commercial development, CC & severe weather: Climate change and severe weather, T & S corridors: Transport and service corridors.

Comparing threats to extant threatened species with drivers of species-level extinctions, it can be seen that agriculture and logging/wood harvesting appear to be more important threats to extant species, while invasive alien species caused more extinctions (Figure 6).

## Discussion

### Known and suspected extinctions

Extinctions have probably been better documented among birds than for any other comparable group of organisms, and indeed more bird species are known to have gone extinct in recent centuries than organisms of any other class (although, proportionally, mammals have suffered a marginally higher extinction rate −15.6% since 1500 vs. 15.0% for birds, comparing totals for species classified as Extinct, Extinct in the Wild and Possibly Extinct [44]). However, even for birds, it is likely that many taxa went extinct before being described to science. On some islands of the western Pacific, cryptic losses of vulnerable species may even exceed the number of species already known from fossils and living birds [45]. It is likely that extinctions of subspecies have been less well documented than those of species, so our totals are almost certainly underestimates. Nevertheless, the total of 138 subspecies (and a total of 279 ultrataxa) we estimate as having gone Extinct or Possibly Extinct since 1500 is a substantial increase in the known loss of avian diversity.

### Timing

Since prehistoric times, humans have been causing avian extinctions [8], [10], [46]–[48]. Due to the paucity of records, we can only guess the magnitude of the wave of destruction following early Polynesians. Based on fossil evidence, around 2,000 species (mostly Rallidae) are thought to have been lost from islands in the Pacific Ocean [46], but most of these pre-dated the post-1500 period considered in our analysis. Exploratory expeditions from Europe in the late 18^th^ century opened the way to remote Pacific islands for European explorers and settlers, who continued and expanded the pressures (human predation, introduction of alien species and landscape alteration) started by Polynesians [6], [49], [50], and drove many taxa extinct that had survived earlier human colonisations. As more extinctions were recorded from recently colonised islands, islands colonised earlier probably lost more species than recorded [34].

The inclusion of subspecies in our analysis revealed a novel feature in the temporal pattern of extinctions. Previous analyses have shown that avian extinctions at the species level peaked in the late 19^th^ century and have declined since, albeit with hints of an increasing wave of continental extinctions [4]. Here we show that at ultrataxon level, the magnitude of this latter phenomenon has accelerated the overall extinction rate since the mid-20^th^ century, and will soon lead to an extinction rate that is unprecedented in recent (post-1500) human history. This reflects the shift from extinction of small-island taxa susceptible to over-exploitation and invasive alien species, to the loss of continental taxa driven by wholesale habitat conversion and degradation. This shift from islands to continents has been predicted to continue, at least for passerines in the Americas, especially those with restricted ranges that occur in areas of high human population and hence intense and multiple pressures [51].

### Taxonomic selectivity

Our analysis supports previous findings that extinctions have not been random with respect to taxonomy [30], [52], [53]. Extinctions have also been disproportionately concentrated in species-poor families, such as Acanthisittidae, Callaeatidae (New Zealand wattlebirds), Dromaiidae, Mohoidae and Raphidae. Such non-random taxonomic distribution of extinction represents a disproportionate loss of genetic variation. Among currently threatened taxa this pattern is not as prominent as it was historically, probably because susceptible taxa have already been lost, and because a higher proportion of threatened taxa are found on continents, where genera and families are more speciose. Some speciose families, such as Anatidae, Columbidae, Fringillidae, Psittacidae and Rallidae have also experienced disproportionately high extinction rates. Some of these taxa possessed traits that made them more extinction prone, including large body size, flightlessness and low rates of fecundity [30], [52], [53]. Large-bodied and terrestrial species were often over-exploited for food (e.g. pigeons), while brightly coloured species were targeted for capture as pets (e.g. parrots) or their body parts used for decoration (e.g. the red and yellow feathers of Hawaiian passerines used as ornaments for royalty [7]). While some families (e.g. Columbidae and Rallidae) with more species threatened than expected have a history of recent extinctions, others have experienced no recent extinctions so far. While Blackburn and Gaston [34] found two families (Corvidae and Zosteropidae) with fewer extinctions than expected at the species level and one family (Meliphagidae) with fewer threatened species than expected, in our simulations the only significant result was that members of the family Timaliidae have experienced fewer extinction than expected at the ultrataxon level. The families (Picidae, Tyrannidae and Paridae) listed by Lockwood et al. [32] as having fewer than the expected number of extinct or threatened species (considered together) all had significant *p* values in our calculations, but the *q* values were not significant.

### Location

The geographic location of subspecific extinctions is similar to that of species, with a substantial proportion lost from oceanic islands (64.4%), and a familiar list of hotspots apparent, such as the Hawaiian Islands, Australia and French Polynesia. It is notable that despite its extraordinarily high levels of avian diversity, South America has experienced only five species-level and seven subspecies-level extinctions. Other continents have similarly few (five in total on mainland Africa and one each on mainland Europe and Asia). This may be because large, species-rich areas such as the Amazon Basin have remained relatively intact until very recently, and because surveying effort has been low relative to the high levels of diversity (it is likely that some restricted-range taxa in the topographically complex Andes were driven extinct before they were described to science). Elsewhere, those regions with long-standing human populations (e.g. Europe and much of Asia) have recorded relatively few extinctions in the past 500 years, suggesting that extinctions of susceptible species may have occurred before 1500 [54].

Similarly, the proportion of the recent avifauna that is now extinct, or endangered on islands is negatively related to the duration of human presence [45], and our documentation of subspecific extinctions further highlights the biodiversity loss that occurred in places such as New Zealand, French Polynesia and the Hawaiian Islands following large-scale human settlement.

### Drivers

Most extinctions since 1500 have been directly or indirectly caused by humans. No species and just one subspecies is known to have been driven extinct by natural catastrophes: the San Benedicto Rock Wren *Salpinctes obsoletus exsul* by a volcanic eruption [55]. Our analysis reaffirms previous findings that invasive alien species, habitat loss driven largely by agricultural expansion, and overexploitation have been the major drivers of extinctions [4], [8], [56], [57], often acting in synergy [58]. The interaction of invasive alien species with habitat loss and habitat degradation is of particular importance [59]. For example, in the Hawaiian Islands, the foraging activities of pigs cause habitat modification, which allows the spread of invasive mosquitoes, which in turn carry Avian Malaria and Avian Pox [7], [60]. Other examples of particularly severe impacts of invasive alien herbivores include Rabbits *Oryctolagus cuniculus* on Macquarie Island [61], domestic Goats *Capra hircus* on Guadalupe [62] and Sheep *Ovis aries* on Mangere Island [63]. Predation, both on adults and eggs or chicks in the nest, is the most important mechanism by which invasive alien species have driven native birds extinct [63], [64], as well as being the most important of their current impact on extant birds [64], [65]. The greatest culprits among predators have been rats *Rattus sp.* [66]–[68], the Cat *Felis catus* [69], [70], and in Guam, the Brown Tree Snake *Boiga irregularis*, which has caused the extinction of three subspecies and a species [71]. Disease caused by introduced pathogens have driven at least 16 species extinct [64].

A comparison of the factors that drove extinctions in the past with the threats that impact extant threatened species reveals that, while the top four factors are the same for both (agriculture-driven habitat loss, logging/wood harvest, over-exploitation and invasive alien species), the first two of these are substantially more significant for extant species, and invasive alien species are reduced from first to fourth most important. The magnitude of habitat loss is increasing at a rate suggesting that agriculture, development and logging/wood harvesting will soon become the leading drivers of extinction.

Climatic, ecological or anthropogenic challenges have already filtered out many of the species that would be most susceptible to existing threats [72]. Some of these threats are still amplifying and reaching new areas [51]. Nevertheless, several new threats have emerged during the last 50 years, such as plastic debris in the oceans affecting seabirds [73] or the large-scale use of pesticides with high avian toxicity [74].

### Preventing human-induced extinctions

Extinction rates would be higher still without conservation efforts [64], [75]–[77], which prevented at least 31 bird species extinctions over the last century [12], [78].

However, the increasing rate of extinctions since the mid-20^th^ century that we document here, and the deteriorating status of extant bird species [64], highlight the increasing scale of the challenge.

An expanding number of species survive only because of constant attention and conservation funding [12], [79]. Such attention increasingly needs to address multiple threatening processes simultaneously in order to avoid cascading effects that can happen when threats are mitigated individually [5]. Finally, the escalating impacts of climate change may soon become the primary driver of biodiversity loss (e.g., [80], [81]). Failure to address these increasing challenges will lead to many more extinctions, impoverishing our planet and reducing the ability of ecosystems to deliver the benefits and services upon which we all ultimately depend.

## Supporting Information

Table S1 **Extinct and Critically Endangered (Possibly Extinct) avian taxa.**
(DOCX)

Table S2 **Avian taxa considered extinct and recognised taxonomically by other authors but not by BirdLife International (2011) for species or Dickinson (2003) for subspecies.** Asterisks indicate four subspecies that are recognised by Dickinson (2003) but were excluded in this study, with explanations given in the notes.
(DOCX)

Table S3 **Avian taxa that have been considered extinct by other authors but are considered extant by this study, either because they have been recently recorded/rediscovered, or because there is insufficient evidence to presume extinction or possible extinction, despite a lack of recent records (see Notes for justifications).**
(DOCX)

Table S4 **The distribution of extinct and threatened taxa across avian families.** L (lower) and U (upper) *q* values indicate the significance of differences from the expected values, i.e. fewer extinctions/lower degree of threat and more extinctions/higher degree of threat than expected, respectively. *Q* values equal zero when none of the simulated values crossed thresholds. Threatened species are those classified as such by BirdLife International (2011) in their IUCN Red List assessment.
(DOCX)

References S1 **References for Tables S1, S2, S3 S4.**
(DOCX)
