# Supplementary material for: Global Patterns and Drivers of Avian Extinctions at the Species and Subspecies Level
Source: PLoS One. 2012 Oct 8;7(10):e47080. doi: 10.1371/journal.pone.0047080 (PMC3466226; doi:10.1371/journal.pone.0047080)
Supplement: Table S1 — Extinct and Critically Endangered (Possibly Extinct) avian taxa. (DOCX) [file pone.0047080.s001.docx]

Table S1.Extinct and Critically Endangered (Possibly Extinct) avian taxa

| Family | Taxon | Former distribution | Extinction date (min.–max. where available)^1^ | Justification notes^2^ | Species / subspecies status^3^ |
| --- | --- | --- | --- | --- | --- |
| **Struthionidae** | Arabian Ostrich *Struthio camelus syriacus* | Middle East | 1966 (1941–1966) | An individual shot and eaten in Bahrain in 1941 is the last confirmed record, but one found dead in Jordan in 1966 is thought to relate to this taxon (Folch 1992). | LC / EX |
| **Dromaiidae** | Tasmanian Emu *Dromaius novaehollandiae diemenensis* | Tasmania, Australia | 1845 | A specimen thought to represent the last wild individual was collected in 1845 (Dove 1924). A captive individual that died in 1873 was probably this subspecies but it is unclear whether this, and another that died in captivity in 1884, were of this taxon or the nominate (Le Souëf 1904, Dickison 1926). | LC / EX |
|  | King Island Emu *Dromaius ater* | King I, Australia | 1804 (1802–1805) | The last wild specimen was collected in 1802, and it was extinct in the wild by 1805. A captive bird survived in France until 1822 (Pfennigwerth 2010). | EX |
|  | Kangaroo Island Emu *Dromaius baudinianus* | Kangaroo I, Australia | 1819 (1802–1836) | It was common when collected in 1802. The last bird was reported to have been killed 'some years before' 1836, when the island was settled permanently (Morgan and Sutton 1836). | EX |
| **Cracidae** | Alagoas Curassow *Mitu mitu* | NE Brazil | 1988 | The most recent reports in the wild were of individuals hunted in 1984 and 1988 (del Hoyo 1994). | EW |
| **Numididae** | Moroccan Guineafowl *Numida meleagris sabyi* | Morocco | 1974 (1950–1974) | Numbers of the subspecies diminished greatly after the 1940s, with the last records from 1970-1974 (Thévenot et al. 2003), although these may refer to hybrids, in which case it may have been extinct as early as 1950 (Martínez 1994). Thorough surveys are required to confirm that the taxon is extinct, and so it is best treated as Critically Endangered (Possibly Extinct). | LC / CR(PE) |
| **Phasianidae** | Italian Grey Partridge *Perdix perdix italica* | Italy | 1982 (1975–1988) | This taxon survived until at least 1975 (Lovari 1975) but was considered extinct by 1988 (Matteucci 1988), as a consequence of hybridisation with captive-bred and released individuals of the nominate subspecies (Liukkonen-Anttila et al. 2002). | LC / EX |
|  | New Mexico Grouse *Tympanuchus phasianellus hueyi* | New Mexico, USA | 1952 | The last specific report of this taxon was from the Colfax County, New Mexico in 1952 (Merrill 1967). | LC / EX |
|  | Heath Hen *Tympanuchus cupido cupido* | NE USA | 1932 | This taxon became restricted to Martha's Vineyard in the 1870s and finally disappeared in 1932 (De Juana 1994). | VU / EX |
|  | New Zealand Quail *Coturnix novaezelandiae* | South I, New Zealand | 1875 | The last record was from around 1875, thought to represent the last individual of the species (Tennyson and Martinson 2006). | EX |
| **Anatidae** | Bering Canada Goose *Branta canadensis asiatica* | Bering Sea region | 1914 | This taxon is considered to have gone extinct around 1914 (Fuller 2000). | LC / EX |
|  | Mauritius Shelduck *Alopochen mauritianus* | Mauritius | 1696 (1693–1698) | By 1693, the species was rare and, by 1698, it was extinct (BirdLife International 2011). | EX |
|  | Reunion Shelduck *Alopochen kervazoi* | Réunion | 1691 (1671–1710) | Dubois mentioned finding geese on the island in 1671–1672, but the species was specifically mentioned to have become extinct by Boucher in 1710(BirdLife International 2011). | EX |
|  | Amsterdam Duck *Anas marecula* | Amsterdam I, French Southern Territories | 1793 | The last report (of presumably this species) was by Barrow in 1793 on St Paul (BirdLife International 2011). | EX |
|  | Mauritius Duck *Anas theodori* | Mauritius | 1696 | The species was last mentioned as extant in 1696 (BirdLife International 2011). | EX |
| **Anatidae** *cont.* | Coues's Gadwall *Anas strepera couesi* | Teraina, Kiribati | 1899 (1874–1924) | The taxon has not been seen since its discovery in 1874 (Carboneras 1992). A 1924 expedition from the Bishop Museum in Honolulu found none (Greenway 1967). | LC / EX |
|  | Borrero's Cinnamon Teal *Anas cyanoptera borreroi* | Colombia | 1955 (1950–1955) | There have been no records since the 1950s and the subspecies is likely to be extinct (Carboneras 1992). More detailed searches would be required to confirm this though, and so it is best considered Critically Endangered (Possibly Extinct). | LC / CR(PE) |
|  | Niceforo's Pintail *Anas georgica niceforoi* | C Colombia | 1951 (1946–1956) | The subspecies was described in 1946 and considered to be extinct by 1956 (Carboneras 1992). | LC / EX |
|  | Labrador Duck *Camptorhynchus labradorius* | NE North America | 1875 | The last confirmed specimen was collected off Long Island, New York, in 1875 (or possibly 1878) (BirdLife International 2011). | EX |
|  | Auckland Islands Merganser *Mergus australis* | Auckland Is, New Zealand | 1906 (1902–1910) | The species was last seen alive in 1902. By the time the first Auckland Islands reserve was set up in 1910, it was extinct (Tennyson and Martinson 2006). | EX |
| **Procellariidae** | Large St Helena Petrel *Pterodroma rupinarum* | St Helena | 1502 | The species presumably became extinct soon after the discovery of the island in 1502 (BirdLife International 2011). | EX |
|  | Jamaica Petrel *Pterodroma caribbaea* | Jamaica | 1936 (1879–1936) | Last collected in 1879 and may have been driven extinct around 1936 due to hunting for food and predation by mongooses (Haynes-Sutton et al. 2009). However, nocturnal petrels are notoriously difficult to detect and without further comprehensive searches the species is best considered Critically Endangered (Possibly Extinct). | CR(PE) |
|  | Small St Helena Petrel *Bulweria bifax* | St Helena | 1502 | The species presumably became extinct soon after the discovery of the island in 1502 (BirdLife International 2011). | EX |
| **Hydrobatidae** | Guadalupe Storm-petrel *Oceanodroma macrodactyla* | Guadalupe, Mexico | 1917 (1912–1922) | The last confirmed report was of a breeding bird seen in 1912. Surveys in 1922 confirmed it is probably extinct (BirdLife International 2011), but because of the difficulties associated with tracking nocturnal petrels, and the suitable conditions that remain on the island, it could survive and is best treated as Critically Endangered (Possibly Extinct). | CR(PE) |
| **Podicipedidae** | Alaotra Grebe *Tachybaptus rufolavatus* | Lake Alaotra, Madagascar | 1997 (1985–2009) | The last confirmed report of the species was in 1985. By 2009, all wetlands where it could feasibly survive had been surveyed, leaving no reasonable doubt that the last individual had died (BirdLife International 2011). | EX |
|  | Atitlan Grebe *Podilymbus gigas* | Lake Atitlán, Guatemala | 1985 (1983–1986) | The species was reduced to 30 individuals by 1983 and was extinct by 1986 (BirdLife International 2011). | EX |
|  | Colombian Grebe *Podiceps andinus* | Lake Tota, Colombia | 1979 (1977–1981) | The last confirmed record was in 1977, and intensive studies in 1981 and 1982 failed to find the species (BirdLife International 2011). | EX |
| **Threskiornithidae** | Reunion Ibis *Threskiornis solitarius* | Mascarenes | 1761 | The last report was by Pingré (1763) who visited Rodrigues in 1761 and reported the bird to be near extinction (BirdLife International 2011). | EX |
| **Ardeidae** | Black-backed Bittern *Ixobrychus novaezelandiae* | South I, New Zealand | 1898 (1890–1900) | The species was last recorded in the 1890s (Tennyson and Martinson 2006) and was extinct by 1900 (BirdLife International 2011). | EX |
|  | Reunion Night-heron *Nycticorax duboisi* | Réunion | 1687 (1674–1700) | The only historical record of the species is by Dubois. By 1700 it was extinct (BirdLife International 2011). | EX |
| **Ardeidae** *cont.* | Mauritius Night-heron *Nycticorax mauritianus* | Mauritius | 1697 (1693–1700) | Leguat's description of "great flights of bitterns" in 1693 probably refers to this species. By 1700 it was extinct (BirdLife International 2011). | EX |
|  | Rodrigues Night-heron *Nycticorax megacephalus* | Rodrigues, Mauritius | 1744 (1726–1761) | Last recorded by Tafforet in 1726. In 1761, Pingré specifically noted that there were no longer any "butors" on the island (BirdLife International 2011). | EX |
|  | Bonin Night-heron *Nycticorax caledonicus crassirostris* | Ogasawara Is, Japan | 1895 (1889–1900) | The last specimen was captured in Ogasawara in 1889. It was extinct by 1900 (Martínez-Vilalta and Motis 1992). | LC / EX |
| **Phalacrocoracidae** | Pallas's Cormorant *Phalacrocorax perspicillatus* | Komandorski Is, Russia | 1852 (1840–1857) | The five known specimens were collected during 1840–1850. In 1882, Stejneger was told by the island's residents that the last birds had disappeared about 30 years before (BirdLife International 2011). | EX |
| **Falconidae** | Guadalupe Caracara *Caracara lutosa* | Guadalupe, Mexico | 1903 | It was last recorded in 1903, and is now extinct (Barton et al. 2004). | EX |
|  | Reunion Kestrel *Falco duboisi* | Réunion | 1672 (1671–1672) | The only historical record of small falcons is of the "*emerillons*" reported by Dubois in 1671–1672 (BirdLife International 2011). | EX |
| **Otididae** | Moroccan Bustard *Ardeotis arabs lynesi* | Morocco | 1962 | The last published record of this taxon was in 1962 (Johnsgard 1991). There have been more recent reports, but these are more likely to refer to *A. a. stieberi* (Collar 1996). Although it is likely to have gone extinct, further searches are needed before confirming this and so it is best treated as Critically Endangered (Possibly Extinct). | LC / CR(PE) |
| **Rallidae** | Hawkins's Rail *Diaphorapteryx hawkinsi* | Chatham Is, New Zealand | 1895 | Recent evidence, including a letter from Dannefarerd to Rothschild in 1895 describing the species's appearance, behaviour and the Moriori hunting method, suggests that this species survived into at least the late 1800s (BirdLife International 2011). | EX |
|  | Red Rail *Aphanapteryx bonasia* | Mauritius | 1693 | The species probably went extinct around the time that Leguat wrote that it was ‘very rare’, in 1693 (Cheke 2006). | EX |
|  | Rodrigues Rail *Aphanapteryx leguati* | Rodrigues, Mauritius | 1744 (1726–1761) | The species was last recorded by Tafforet in 1726. In 1761, Pingré noted that it was extinct (BirdLife International 2011). | EX |
|  | Goldman's Rail *Coturnicops noveboracensis goldmani* | Rio Lerma, Mexico | 1964 | The taxon has not been seen since 1964 and much of its wetland habitat has been drained. However, there is a possibility that it may survive (Taylor 1996), so until further surveys have been made it is best treated as Critically Endangered (Possibly Extinct). | LC / CR(PE) |
|  | Bar-winged Rail *Nesoclopeus poecilopterus* | Fiji | 1973 | It is known from 12 specimens from Vitu Levu and Ovalau collected in the 19^th^ century, and reports from Taveuni in 1971 and Vitu Levu in 1973 (BirdLife International 2011). | EX |
|  | Macquarie Rail *Gallirallus philippensis macquariensis* | Macquarie I, Australia | 1887 (1879–1994) | The taxon was common in 1879 (Scott 1882) but could not be found in 1894 (Hamilton 1894) or subsequently. | LC / EX |
|  | Guam Rail *Gallirallus owstoni* | Guam | 1987 | The species was extirpated from the wild in 1987 (BirdLife International 2011). | EW |
|  | Wake Island Rail *Gallirallus wakensis* | Wake I, United States Minor Outlying Islands | 1946 (1945–1946) | It has not been seen since 1945. Thomas D. Musson, a resident of the island from 1946-1949, never saw the species (Greenway 1967). | EX |
|  | Tahiti Rail *Gallirallus pacificus* | Society Is, French Polynesia | 1939 | It was last recorded from Mehetia in the 1930s (Taylor and Van Perlo 1998). | EX |
| **Rallidae** *cont.* | Dieffenbach's Rail *Gallirallus dieffenbachii* | Chatham Is, New Zealand | 1856 (1840–1872) | Already scarce when the type specimen was collected in 1840, the species was extinct by 1872 (BirdLife International 2011). | EX |
|  | Chatham Rail *Cabalus modestus* | Chatham I, New Zealand | 1894 (1893–1895) | The last confirmed live birds were found in 1893. It was extinct by 1895 (Tennyson and Martinson 2006). | EX |
|  | Peruvian Rail *Rallus semiplumbeus peruvianus* | Peru | 1886 | It is known only from the type specimen, collected in 1886 (Taylor 1996). This taxon is likely to have gone extinct, but as it is so poorly known, shy and cryptic, and lives in thick forest, this cannot yet be confirmed, and it is best considered Critically Endangered (Possibly Extinct). | EN / CR(PE) |
|  | Western Lewin's Rail *Lewinia pectoralis clelandi* | Southwest Australia | 1955 (1932–1977) | This taxon has not been seen since 1932 (Taylor 1996), with a specimen collected in 1931 (Whittell 1933). Atlas surveys in 1977–1981 failed to locate it (Blakers et al. 1984), as did searches of its wetland range in 1981–1985 and follow up atlas work in 1998–2002 (Barrett et al. 2003). | LC / EX |
|  | Assumption Rail *Dryolimnas cuvieri abbotti* | Assumption, Seychelles | 1923 (1908–1937) | The taxon was still abundant in 1908 (Fryer 1911), but extinct by 1937 (Taylor and Van Perlo 1998). | LC / EX |
|  | Ascension Crake *Mundia elpenor* | Ascension I, St Helena | 1656 | The only record comes from 1656. It probably went extinct due to predation by introduced rats (*Rattus* spp.) (Ashmole 1963). | EX |
|  | St Helena Crake *Atlantisia podarces* | St Helena | 1502 | The species presumably became extinct soon after the discovery of the island in 1502 (BirdLife International 2011). | EX |
|  | Jamaican Crake *Amaurolimnas concolor concolor* | Jamaica | 1881 | The taxon is thought to have declined rapidly following Small Asian Mongoose (*Herpestes javanicus*) introduction in 1872, and was last recorded in Jamaica in 1881 (Taylor 1996). | LC / EX |
|  | Miller's Rail *Porzana nigra* | Tahiti, French Polynesia | 1784 | The species is known from illustrations by Miller from 1784 (although these are sometimes considered to refer to Spotless Crake), and it presumably became extinct soon after this (BirdLife International 2011). | EX |
|  | St Helena Rail *Porzana astrictocarpus* | St Helena | 1502 | The species presumably became extinct soon after the discovery of the island in 1502 (BirdLife International 2011). | EX |
|  | Laysan Rail *Porzana palmeri* | NW Hawaiian Is, USA | 1944 | The population on Laysan became extinct between 1923 and 1936. Black rats (*Rattus rattus*), brought in by wartime activities, exterminated the introduced populations on Sand Island in 1943 and Eastern Island in 1944 (BirdLife International 2011). | EX |
|  | Hawaiian Rail *Porzana sandwichensis* | Hawaiian Is, USA | 1884 | The last specimen was collected in 1864, and the last report was in 1884 (or possibly 1893) (BirdLife International 2011). | EX |
|  | Kosrae Crake *Porzana monasa* | Kosrae, Micronesia | 1853 (1827–1878) | The only two known specimens were collected in 1827–1828. The species declined to extinction over the next half-century following the arrival of rats (*Rattus* spp.) from missionary and whaling ships in the 1830s and 1840s (BirdLife International 2011). | EX |
|  | Reunion Gallinule *Porphyrio coerulescens* | Réunion | 1730 | Extinct by around 1730. Known only from travellers’ descriptions (BirdLife International 2011). | EX |
|  | New Caledonia Gallinule *Porphyrio kukwiedei* | Grande Terre, New Caledonia | 1860 | The only historical record of the species is a passage from Verreaux and Des Murs written in 1860, noting the presence of birds the size of turkeys in marshy areas (BirdLife International 2011). | EX |
| **Rallidae** *cont.* | White Gallinule *Porphyrio albus* | Lord Howe I, Australia | 1812 (1790–1834) | Not uncommon when discovered in 1790, this species had probably already vanished by the time the island was colonised in 1834 (BirdLife International 2011). | EX |
|  | North Island Takahe *Porphyrio mantelli* | North I, New Zealand | 1894 | The species is known from subfossils from a number of archaeological sites, and from one possible 1894 record (BirdLife International 2011). | EX |
|  | Tristan Island Cock *Gallinula nesiotis nesiotis* | Tristan da Cunha, St Helena | 1890 (1873–1906) | Reports from the Challenger expedition of 1873 suggest it was extant (Beintema 1972), but villagers contacted on the Valhalla expedition in 1906 said it was no longer present (Nicoll 1906). | VU / EX |
|  | Mascarene Coot *Fulica newtoni* | Mascarenes | 1693 | Last reported on Réunion by Dubois in 1672, and on Mauritius by Leguat in 1693 (BirdLife International 2011). | EX |
| **Turnicidae** | New Caledonia Buttonquail *Turnix varius novaecaledoniae* | Grande Terre, New Caledonia | 1930 (1912–1947) | The last known individual was taken by Saracin in 1912. Warner's surveys were unsuccessful (Warner 1947), as was more recent fieldwork (Ekstrom et al. 2002), though as neither were comprehensive it is best considered Critically Endangered (Possibly Extinct). | LC / CR(PE) |
| **Haematopodidae** | Canary Islands Oystercatcher *Haematopus meadewaldoi* | E Canary Is, Spain | 1945 (1913–1950) | The species was last collected in 1913 and locally reported to have become extinct by the 1940s (BirdLife International 2011). | EX |
| **Scolopacidae** | Stewart Snipe *Coenocorypha aucklandica iredalei* | Stewart I, New Zealand | 1964 | Only two snipe were found after the accidental introduction of black rats (*Rattus rattus*) onto the subspecies's final refuge on Stewart Island in 1964. Both were taken into captivity but died soon after (Tennyson and Martinson 2006). | NT / EX |
|  | Barrier Snipe *Coenocorypha aucklandica barrierensis* | Little Barrier I, New Zealand | 1875 (1870–1880) | The taxon is known only from a specimen collected in 1870 (Piersma et al. 1996). It is thought to have gone extinct during the 1870s (Tennyson and Martinson 2006). | NT / EX |
|  | Eskimo Curlew *Numenius borealis* | Americas | 1963 | The last irrefutable record is of a specimen collected in Barbados in 1963. There have been numerous unconfirmed reports since, especially between 1981 and 2006, and so the species is best considered Critically Endangered (Possibly Extinct) (BirdLife International 2011). | CR(PE) |
|  | White-winged Sandpiper *Prosobonia ellisi* | Moorea, French Polynesia | 1777 | Known only from a specimen collected in 1777, the species was presumably was driven to extinction by introduced rats (*Rattus* spp.) in the late 18th century (BirdLife International 2011). | EX |
|  | Kiritimati Sandpiper *Prosobonia cancellata cancellata* | Kiritimati, Kiribati | 1831 (1789–1873) | The taxon is only known from its first recording in 1789 (Walters 1993). The first thorough ornithological survey of Kiritimati, in 1873–1875, found none (Streets 1877). | EN / EX |
|  | Tahitian Sandpiper *Prosobonia leucoptera* | Tahiti, French Polynesia | 1777 | The last specimen, now lost, was taken by Forster and Anderson in 1777 (Greenway 1967). | EX |
| **Alcidae** | Great Auk *Pinguinus impennis* | N Atlantic | 1852 | The last known pair were killed on Eldey I, Iceland, in 1844, and the last live bird was seen off the Newfoundland Banks in 1852 (BirdLife International 2011). | EX |
| **Pteroclididae** | Fayyum Sandgrouse *Pterocles exustus floweri* | Fayyum area, Egypt | 1995 (1979–1995) | The last sighting was of 10 birds between Isna and Idfu in March 1979 (De Juana 1997) and the species is likely to have gone extinct since. However, further searches are required to confirm this, and the taxon is best considered Critically Endangered (Possibly Extinct). | LC / CR(PE) |
| **Raphidae** | Dodo *Raphus cucullatus* | Mauritius | 1662 | The species probably went extinct on mainland Mauritius in the early 1930s (Mlíkovský 2004), but the last record is of birds killed on Ile d’Ambre in 1662. These were probably the last individuals of the species (Cheke 2006). | EX |
|  | Rodrigues Solitaire *Pezophaps solitaria* | Rodrigues, Mauritius | 1770 (1761–1778) | Pingré was informed of the birds' presence in 1761, but Morel reported in 1778 that they were certainly extinct (Cheke 1987). | EX |
| **Columbidae** | St Helena Dove *Dysmoropelia dekarchiskos* | St Helena | 1502 | The species presumably became extinct soon after the discovery of the island in 1502 (BirdLife International 2011). | EX |
|  | Reunion Pigeon *Columba duboisi* | Réunion | 1674 | The species was described in 1674 and is likely to have gone extinct in the early 1700s (BirdLife International 2011). | EX |
|  | Madeiran Wood-pigeon *Columba palumbus maderensis* | Madeira, Portugal | 1912 (1900–1924) | The taxon’s extinction is dated as early 20th century by Fuller (2000). None were found in a survey in May 1924 (Meinertzhagen 1925). | LC / EX |
|  | Lord Howe Pigeon *Columba vitiensis godmanae* | Lord Howe I, Australia | 1861 (1853–1869) | This subspecies is recognised here *contra* Dickinson (2003), following Schodde and Mason (1997). It was last recorded in 1853 and almost certainly extinct by 1869 (McAllan et al. 2004). | LC / EX |
|  | Bonin Wood-pigeon *Columba versicolor* | Ogasawara Is, Japan | 1889 | The species was last recorded in 1889 (BirdLife International 2011). | EX |
|  | Ryukyu Pigeon *Columba jouyi* | Ryukyu Is, Japan | 1936 | It was last seen on the Daito Islands in 1936. In the 1960s it was believed to possibly survive on isolated islets, but it there have been no further sightings and it is clearly extinct (Brazil 1991). | EX |
|  | Amirante Turtle-dove *Nesoenas picturata aldabrana* | Amirante Is, Seychelles | 1974 | Small numbers persisted until at least 1974, but the population has since been swamped through hybridisation with the introduced nominate subspecies (Baptista et al. 1997). It is now considered extinct (Skerrett and Disley 2011). | LC / EX |
|  | Passenger Pigeon *Ectopistes migratorius* | N America | 1905 (1900–1910) | The last reliable specimen came from Ohio in 1900. A two-year search was carried out beginning in 1910, to determine whether the species was extinct in the wild. It found no evidence for its persistence (Blockstein and Tordoff 1985). The last captive bird died in 1914 in the Cincinnati Zoo (BirdLife International 2011). | EX |
|  | Socorro Dove *Zenaida graysoni* | Socorro, Mexico | 1972 | The last sighting in the wild was in 1972 (BirdLife International 2011). | EW |
|  | Liverpool Pigeon *Caloenas maculata* | Tahiti, French Polynesia | 1928 (1783–1928) | The species is only known from the type specimen, collected sometime between 1783 and 1823. It is thought to have come from Tahiti, based on local accounts from 1928 which match the description of the species (BirdLife International 2011). | EX |
|  | Norfolk Island Ground-dove *Gallicolumba norfolciensis* | Norfolk Island | 1820 (1800–1840) | The species was present in 1800 (Hindwood 1965), but not detected in 1840 (Moore 1985). | EX |
|  | Tuamotu Ground-dove *Gallicolumba erythroptera pectoralis* | S Tuamotu Is, French Polynesia | 1961 (1922–1999) | This subspecies is recognised following Gibbs et al. (2001) and *contra* Dickinson (2003) because the type specimens considered sufficiently distinctive to warrant subspecies status by BirdLife International. In 1922, a specimen was collected at Tuanake and six at Hiti (Holyoak and Thibault 1984). It is treated as Critically Endangered (Possibly Extinct) because of uncertainty over the taxonomic affinity of some extant birds, but seems to have not been recorded since, including searches in 1999 (Blanvillain et al. 2002). | CR / CR(PE) |
|  | Tanna Ground-dove *Gallicolumba ferruginea* | Tanna, Vanuatu | 1774 | Known only from Forster's 1774 painting (BirdLife International 2011). | EX |
|  | Thick-billed Ground-dove *Gallicolumba salamonis* | Makira, Solomon Islands | 1961 (1927–1995) | The species has not been recorded since a specimen was taken in 1927. Surveys, the first of which was in 1995, have been unsuccessful in relocating it (BirdLife International 2011). | EX |
|  | Choiseul Pigeon *Microgoura meeki* | Choiseul, Solomon Islands | 1904 | The species was last recorded in 1904. Reports from the 1940s are not thought to be accurate. It is now considered Extinct (BirdLife International 2011). | EX |
| **Columbidae** *cont.* | Hiva Oa Red-moustached Fruit-dove *Ptilinopus mercierii tristrami* | Hiva Oa, French Polynesia | 1922 | At least 11 specimens are known, the last of which was collected by the Whitney Expedition in 1922 (Holyoak and Thibault 1984). Reports from Hiva Oa in 1980 were presumably mistaken (Collar et al. 1994). | EX / EX |
|  | Nuku Hiva Red-moustached Fruit-dove *Ptilinopus mercierii mercierii* | Nuku Hiva, French Polynesia | 1849 (1836–1922) | The taxon is known only from the type specimen collected during the Venus voyage of 1836–1839, and was not found during the Whitney Expedition of 1922 (Holyoak and Thibault 1984). Its date of extinction is estimated at 1849 (A. Gouni*in litt.* 2011). | EX / EX |
|  | Rodrigues Blue-pigeon *Alectroenas rodericana* | Rodrigues, Mauritius | 1726 | It was last recorded by Tafforet in 1726 (Cheke 1987). | EX |
|  | Mauritius Blue-pigeon *Alectroenas nitidissima* | Mauritius | 1835 (1832–1837) | It was reported to still be present by Desjardins (1832), but was extinct by the mid-1930s (Cheke 1987). | EX |
|  | Negros Spotted Imperial-pigeon *Ducula carola nigrorum* | Visayas, Philippines | 1970 (1950–1991) | The taxon was reported as common on Negros as recently as the 1950s (Ripley and Rabor 1956). However, it was not found in surveys of the island in 1991 (Brooks et al. 1992, Evans et al. 1993). It is likely to also be extinct on Siquijor (BirdLife International 2011), but further surveys are required to confirm this and so it is best treated as Critically Endangered (Possibly Extinct). | VU / CR(PE) |
|  | Norfolk Island Pigeon *Hemiphaga novaeseelandiae spadicea* | Norfolk Island | 1904 (1900–1908) | It was last recorded in 1900 (Schodde et al. 1983), and not seen in a 1908 survey (Hull 1909). | NT / EX |
| **Psittacidae** | Norfolk Island Kaka *Nestor productus* | Norfolk Island | 1870 | The species went extinct in the wild in the mid-19th century on Norfolk Island, although it may have persisted a little longer on nearby Phillip Island. The last recorded living bird was in captivity in London in 1851(Garnett et al. 2011). It was not recorded on searches of the island in 1908 (Hull 1909). | EX |
|  | Siquijor Hanging-parrot *Loriculus philippensis siquijorensis* | Siquijor, Philippines | 1950 (1900–2000) | The taxon disappeared during the 20^th^ century (Fuller 2000). Claims from the early 1990s suggest it may survive although they are probably more likely to refer to a different race introduced by the pet trade. This slight uncertainty means it is better considered Critically Endangered (Possibly Extinct). | LC / CR(PE) |
|  | Rodrigues Parrot *Necropsittacus rodericanus* | Rodrigues, Mauritius | 1761 | Last reported in 1763 based on observations from 1761, the species was presumably hunted to extinction soon after (BirdLife International 2011). | EX |
|  | Sangihe Red-and-blue Lory *Eos histrio histrio* | Sangihe Is, Indonesia | 1999 (1985–1999) | Was 'very uncommon' in 1985 (Nash 1993). There were many local reports in the mid-1990s, but by 1999 locals thought it gone from the last remaining site and 10 months’ worth of fieldwork had failed to find it (BirdLife International 2001). The race is now thought to be probably extinct (BirdLife International 2011), but is best treated as Critically Endangered (Possibly Extinct) as some uncertainty remains. | EN / CR(PE) |
|  | Raiatea Parakeet *Cyanoramphus ulietanus* | Raiatea, French Polynesia | 1773 | Known from two specimens collected in 1773 (BirdLife International 2011). | EX |
|  | Macquarie Parakeet *Cyanoramphus novaezelandiae erythrotis* | Macquarie I, Australia | 1892 (1890–1894) | In 1894 no birds had been reported for two years. The last definitive record seems to be an observation in 1890 (Taylor 1979). Surveys by Hamilton (1894) found none. | VU / EX |
|  | Lord Howe Parakeet *Cyanoramphus novaezelandiae subflavescens* | Lord Howe I, Australia | 1870 (1869–1887) | One pair was collected in 1869. Consensus is that the taxon went extinct around 1870 (Hindwood 1940) and it was not recorded in surveys in 1887 (Etheridge 1888) or subsequently. | VU / EX |
| **Psittacidae** *cont.* | Black-fronted Parakeet *Cyanoramphus zealandicus* | Tahiti, French Polynesia | 1844 | The last specimen was collected by de Marolles in 1844 (Voisin and Voisin 1995). | EX |
|  | Paradise Parrot *Psephotus pulcherrimus* | E Australia | 1928 | Photographed at the nest in 1927, the last observation was in 1928 (Olsen 2008). Repeated searches since have found none. | EX |
|  | Mascarene Parrot *Mascarinus mascarinus* | Réunion | 1790 (1775–1804) | The last accounts of wild birds were from 1775, and birds were not mentioned by Bory in 1804, so it may have been hunted to extinction in the wild by then. A captive bird survived in the King of Bavaria's menagerie until at least 1834 (BirdLife International 2011). | EX |
|  | Seychelles Parakeet *Psittacula wardi* | Seychelles | 1900 (1893–1906) | It was last recorded on Mahé in 1893, when Abbott shot a specimen (Skerrett and Disley 2011), and extinct by 1906 when Nicoll visited the islands (BirdLife International 2011). | EX |
|  | Newton's Parakeet *Psittacula exsul* | Rodrigues, Mauritius | 1876 | The last record was of a bird procured from locals in 1875. It is thought that the last few individuals were wiped out by intense cyclones in 1876 (Cheke 1987). | EX |
|  | Mauritius Grey Parrot *Lophopsittacus bensoni* | Mascarenes | 1764 | The species was last reported in 1764 (BirdLife International 2011). | EX |
|  | Broad-billed Parrot *Lophopsittacus mauritianus* | Mauritius | 1680 (1673–1693) | Hoffman gave the last definite reports of the species based on observations in 1673–1675. They probably died out around 1680, and the species was not mentioned in reports from 1693 or later (Cheke 1987). | EX |
|  | Spix's Macaw *Cyanopsitta spixii* | Bahia, Brazil | 2000 | The last known individual in the wild disappeared at the end of 2000. However, it cannot yet be presumed to be extinct in the wild until all possible habitat has been surveyed (BirdLife International 2011). | CR(PEW) |
|  | Jamaican Red Macaw *Ara gossei* | Jamaica | 1800 (1765–1800) | A specimen was taken around 1765, and the species was presumably hunted to extinction around the end of the 18th century (BirdLife International 2011). | EX |
|  | Dominican Green-and-yellow Macaw *Ara atwoodi* | Martinique | 1800 | Known from the writings of Atwood in 1791, the species presumably went extinct in the late 18^th^ or early 19^th^ century (BirdLife International 2011). | EX |
|  | Jamaican Green-and-yellow Macaw *Ara erythrocephala* | Jamaica | 1810 | Described in 1847, the species was presumably hunted to extinction in the early 19^th^ century (BirdLife International 2011). | EX |
|  | Lesser Antillean Macaw *Ara guadeloupensis* | Guadeloupe | 1760 | It was rare by 1760, and presumably went extinct soon after (BirdLife International 2011). | EX |
|  | Cuban Macaw *Ara tricolor* | Cuba | 1885 | The species was last collected in 1864, with the last reports in 1885. A number of other related species (additional to those mentioned here) have been described from the West Indies, but are not based on sound evidence (BirdLife International 2011). | EX |
|  | Guadeloupe Parakeet *Aratinga labati* | Guadeloupe | 1775 | Probably declined to extinction due to hunting in the second half of the 18^th^ century. Only known from descriptions, the former existence of this bird is plausible biogeographically and because described details cannot refer to other known species (BirdLife International 2011). | EX |
|  | Puerto Rican Conure *Aratinga chloroptera maugei* | Mona I, Puerto Rico | 1900 | The taxon went extinct around 1900 (Fuller 2000). | VU / EX |
| **Psittacidae** *cont.* | Sinu Brown-throated Parakeet *Aratinga pertinax griseipecta* | Sinú Valley, Colombia | 1949 | The taxon was last seen and collected from Tierralta in 1949 and is probably extinct. However, further surveys would be required to confirm this (T. M. Donegan *in litt.* 2011) and it is best treated as Critically Endangered (Possibly Extinct). | LC / CR(PE) |
|  | South-eastern Parakeet *Conuropsis carolinensis carolinensis* | Florida region, USA | 1904 | The last specimens were collected by Chapman near Lake Okeechobee, Florida, in 1904, although rumours of its survival persisted into the 1930s (McKinley 1985). The last captive bird died in the Cincinnati Zoo in 1918 (Joines 1985). | EX / EX |
|  | Louisiana Parakeet *Conuropsis carolinensis ludoviciana* | Louisiana region, USA | 1910 | The last bird of this subspecies is thought to have died around 1910 (BirdLife International 2011). | EX / EX |
|  | Sinu Painted Parakeet *Pyrrhura picta subandina* | Sinú Valley, Colombia | 2005 | The taxon was not considered extinct by Joseph and Stockwell (2002), although they express concern for its survival. Its known range was searched without success in 2005–2008 (P. Salaman *in litt.* 2011) and surveys elsewhere have also not found it (Stiles et al. 1999). It is thus now likely to be extinct, but further searches of its possible range are required to confirm this, so it is best treated as Critically Endangered (Possibly Extinct). | LC / CR(PE) |
|  | Guadeloupe Amazon *Amazona violacea* | Guadeloupe | 1779 | Noted by Buffon in 1779 to be very rare, the species presumably became extinct soon after (BirdLife International 2011). | EX |
|  | Martinique Amazon *Amazona martinicana* | Martinique | 1790 (1779–1800) | The species was noted by Buffon in 1779, but it declined very rapidly to extinction in the latter half of the 18^th^ century (BirdLife International 2011). | EX |
|  | Culebra Island Amazon *Amazona vittata gracilipes* | Culebra, Puerto Rico | 1912 (1899–1912) | Baker collected three specimens in 1899 (Forshaw 1989). The subspecies supposedly became extinct in 1912 (BirdLife International 2011). | CR / EX |
| **Cuculidae** | St Helena Cuckoo *Nannococcyx psix* | St Helena | 1750 | The species presumably went extinct as a result of the deforestation of the island in the 18^th^ century (BirdLife International 2011). | EX |
|  | Snail-eating Coua *Coua delalandei* | Madagascar | 1834 | It has not been reported since 1834 (Morris and Hawkins 1998). | EX |
|  | Assumption Island Coucal *Centropus toulou assumptionis* | Assumption, Seychelles | 1906 | The last record seems to be from 1906 (Nicoll 1906). | LC / EX |
|  | Cabo San Lucas Groove-billed Ani *Crotophaga sulcirostris pallidula* | Baja California, Mexico | 1921 | Described in the early 20^th^ century (Bangs and Pernard 1921) and probably went extinct at a similar time. However, the taxon is very poorly known. | LC / CR(PE) |
| **Strigidae** | Virgin Islands Screech-owl *Megascops nudipes newtoni* | Virgin Islands | 1953 (1927–1979) | Wetmore was informed of its presence on Vieques by locals in 1927 (Wetmore 1927). There have been many unconfirmed reports since (Leck 1975, Nellis 1979, Norton 1986), but surveys, the first in 1979, have been unsuccessful (Moreno 1998, Marks et al. 1999). Extinction cannot be confirmed because of the number of reports and the difficulties in detecting this small, nocturnal species, so it is best considered Critically Endangered (Possibly Extinct). | LC / CR(PE) |
|  | Reunion Owl *Mascarenotus grucheti* | Réunion | 1610 | The species presumably became extinct soon after the island's colonisation in the early 17^th^ century (BirdLife International 2011). | EX |
| **Strigidae** *cont.* | Rodrigues Owl *Mascarenotus murivorus* | Rodrigues, Mauritius | 1726 | It was last recorded by Tafforet in 1726 (Cheke 1987). | EX |
|  | Mauritius Owl *Mascarenotus sauzieri* | Mauritius | 1848 (1837–1859) | The last reports were in 1837 and Clark specifically wrote that it was extinct in 1859 (BirdLife International 2011). | EX |
|  | Socorro Elf Owl *Micrathene whitneyi graysoni* | Socorro, Mexico | 1946 (1931–1960) | The taxon was last collected in1931 (Jehl and Parkes 1982). It was not seen in 1960 and most available habitat has been surveyed without success, including in 1978 and 1993 (Wehtje et al. 1993). However it is tiny, nocturnal and cryptic and so may have been missed. For these reasons it is considered Critically Endangered (Possibly Extinct). | LC / CR(PE) |
|  | Guadeloupe Burrowing Owl *Athene cunicularia guadeloupensis* | Guadeloupe | 1890 | The taxon went extinct around 1890 (Marks et al. 1999). | LC / EX |
|  | Antiguan Burrowing Owl *Athene cunicularia amaura* | Antigua | 1890 (1890–1903) | The taxon went extinct around 1890 (Marks et al. 1999), when Winch collected specimens. None were found in Branch's 1903 survey (Danforth 1934). | LC / EX |
|  | Lord Howe Island Boobook*Ninox novaeseelandiae albaria* | Lord Howe I, Australia | 1947 (1922–1972) | The pure form of this subspecies was certainly present in 1908 (Hull 1909). Conspecifics probably began hybridising soon after their introduction in 1922. The species was last heard on Lord Howe in the 1950s (McAllan et al. 2004) and surveys of the island's avifauna in the 1970s found none (Disney and Smithers 1972, Recher and Clark 1974). | LC / EX |
|  | South Island Laughing Owl *Sceloglaux albifacies albifacies* | South I, New Zealand | 1914 | The last confirmed record was from 1914 (Tennyson and Martinson 2006). | EX / EX |
|  | Rufous-faced Laughing Owl *Sceloglaux albifacies rufifacies* | North I, New Zealand | 1890 | The taxon is thought to have gone extinct around 1890 (A. J. D. Tennyson *in litt.* 2011). | EX / EX |
| **Caprimulgidae** | New Caledonian Nightjar *Eurostopodus mystacalis exul* | Grande Terre, New Caledonia | 1939 | The only known record is a single specimen taken in 1939. It is probably extinct (Dutson 2011), but as it is so poorly known it is best treated as Critically Endangered (Possibly Extinct). | LC / CR(PE) |
|  | Jamaican Pauraque *Siphonorhis americana* | Jamaica | 1860 | The species was last recorded in 1860. Reports of unidentifiable nightjars from its habitat in the 1980s have led to suggestions that may still be extant, and there is a possibility that surveys may have overlooked this cryptic, nocturnal species (BirdLife International 2011). Therefore it is best considered Critically Endangered (Possibly Extinct). | CR(PE) |
| **Trochilidae** | Gould's Emerald *Chlorostilbon elegans* | Jamaica | 1860 | The species is known only from the type specimen collected in 1860 (BirdLife International 2011). | EX |
|  | Brace's Emerald *Chlorostilbon bracei* | New Providence, Bahamas | 1877 | The species is only known from the type specimen, which Brace collected in 1877. It is likely to have gone extinct around this time, because collectors visiting the island soon afterwards found no trace of it (Graves and Olson 1987). | EX |
|  | Miravalles Hummingbird *Amazilia cyanifrons alfaroana* | Costa Rica | 1895 | The only record comes from an expedition to Costa Rica in 1895 (Underwood 1896) and it is likely to be extinct, but further searches are required to confirm this. It is therefore best treated as Critically Endangered (Possibly Extinct). | LC / CR(PE) |
|  | Leybold's Fire-crown *Sephanoides fernandensis leyboldi* | Alejandro Selkirk I, Chile | 1908 | It was last recorded on the island in 1908 (Schuchmann 1999, BirdLife International 2011). | CR / EX |
| **Trochilidae** *cont.* | Turquoise-throated Puffleg *Eriocnemis godini* | Ecuador | 1915 (1850–1980) | The species has not been recorded since the 19th century, with an unconfirmed report in 1976 and no subsequent records despite a specific search in 1980 (BirdLife International 2011). The habitat at the type locality has been destroyed, but the distribution of the species is uncertain. It is likely to be extinct, but further searches are required to confirm this. It is therefore best treated as Critically Endangered (Possibly Extinct). | CR(PE) |
| **Alcedinidae** | Guam Kingfisher *Todiramphus cinnamominus cinnamominus* | Guam | 1986 | The taxon has been extinct in the wild since 1986, when 29 birds were taken for captive breeding (Woodall 2001). | LC / EW |
|  | Mangareva Kingfisher *Todiramphus gambieri gambieri* | Mangareva, French Polynesia | 1922 | The taxon became extinct around 1922 (Thibault 1973, Holyoak and Thibault 1977, 1984, Seitre and Seitre 1991, 1992, Woodall 2001). A report from the 2000s is not considered to be accurate (P. Raust *in litt.* 2011). | CR / EX |
|  | Sangihe Kingfisher *Ceyx fallax sangirensis* | Sangihe I, Indonesia | 1997 | The taxon has not been recorded since 1997 (Riley 2002); this author now considers the subspecies extinct, as the extant *C. f. fallax* is easy to observe on Sulawesi (J. Riley *in litt.* 2012). However the latest record is recent enough for it to be considered Critically Endangered (Possibly Extinct) here. | NT / CR(PE) |
| **Upupidae** | St Helena Hoopoe *Upupa antaios* | St Helena | 1502 | The species was presumably hunted to extinction by people and introduced predators soon after the discovery of the island in 1502 (BirdLife International 2011). | EX |
| **Bucerotidae** | Ticao Hornbill *Penelopides panini ticaensis* | Ticao, Philippines | 1949 (1905–1993) | The taxon was abundant at the start of the century (McGregor 1905), but there have been no recent records. An expedition to Ticao in 1993 did not record any, and interviews with locals gave no suggestion of its persistence (Curio 1994). Little forest remains where it could survive (Kemp 2001). | EN / EX |
| **Picidae** | Guadalupe Flicker *Colaptes auratus rufipileus* | Guadalupe, Mexico | 1906 | The subspecies was last recorded in 1906, when the population was estimated at 40 birds of which 12 were collected (Winkler and Christie 2002). It presumably went extinct soon after. | LC / EX |
|  | Cebu White-bellied Woodpecker *Dryocopus javensis cebuensis* | Cebu, Philippines | 2001 | This subspecies is generally accepted as extinct, although the date of its last record is unclear. A three-year survey in the early 2000s found none (Paguntalan and Jakosalem 2008). | LC / EX |
|  | Imperial Woodpecker *Campephilus imperialis* | Durango, Mexico | 1956 (1956–1995) | The last confirmed record was from Durango in 1956 but there have been several convincing local reports of sightings since 1965.Although it has been searched for extensively, it remains possible that individuals persist (BirdLife International 2011) and so it is best treated as Critically Endangered (Possibly Extinct). | CR(PE) |
|  | Cuban Ivory-billed Woodpecker *Campephilus principalis bairdii* | Cuba | 1992 (1991–1992) | An expedition in 1991 found only a single bird, and fieldwork in 1992 and 1993 failed to locate any in eastern Cuba. There are a few patches of unsurveyed habitat though, and there was an unconfirmed report in 1998 (Winkler and Christie 2002). In light of this the taxon is best considered Critically Endangered (Possibly Extinct). | CR / CR(PE) |
|  | Northern Ivory-billed Woodpecker *Campephilus principalis principalis* | SE USA | 1980 (1950–2009) | The last confirmed records came from 1950. There have been recent unconfirmed claims of the taxon’s existence in Arkansas and Florida, but intensive follow-up searches, notably in 2009, have failed to find conclusive evidence (BirdLife International 2011) suggesting it is likely to have gone extinct. Further searches would be needed to confirm this though, and it is therefore considered Critically Endangered (Possibly Extinct). | CR / CR(PE) |
| **Acanthisittidae** | South Island Bush Wren *Xenicus longipes longipes* | South I, New Zealand | 1972 | The taxon was last recorded in 1972 (Collar et al. 1994). | EX / EX |
|  | Stead's Bush Wren *Xenicus longipes variabilis* | Titi Is, New Zealand | 1965 | The taxon was last recorded in 1965 (Collar et al. 1994). | EX / EX |
|  | North Island Bush Wren *Xenicus longipes stokesi* | North I, New Zealand | 1911 | The taxon went extinct in 1911 (A. J. D. Tennyson *in litt.* 2011). | EX / EX |
| **Acanthisittidae** *cont.* | Stephens Island Wren *Traversia lyalli* | Stephens I, New Zealand | 1895 | The species was extinct by November 1895, but it had survived into that year, with Travers finding a cat-caught specimen in February (Tennyson and Martinson 2006). | EX |
| **Tyrannidae** | Bogota Tachuri *Polystictus pectoralis bogotensis* | Bogotá area, Colombia | 1959 (1950–2004) | The taxon has not been reliably recorded since specimens were taken in the 1950s (Collar and Wege 1995), with one possible more recent sighting from the Bogotá area (F. G. Stiles *in litt.* 1994). It was considered Extinct by Donegan (2004), who now suggests it is "long gone" (T. M. Donegan *in litt.* 2011). | NT / EX |
|  | Grenadan Euler's Flycatcher *Lathrotriccus euleri flaviventris* | Grenada | 1950 | Not recorded since 1950 (Fitzpatrick et al. 2004).However, surveys have not been comprehensive (D.C. Wege *in litt.* 2011) and until further work is carried out it is best treated as Critically Endangered (Possibly Extinct). | LC / CR(PE) |
|  | San Cristobal Vermilion Flycatcher *Pyrocephalus rubinus dubius* | Galápagos Is, Ecuador | 1992 (1980–1998) | The last sight record was in the 1980s and it has since been lost on San Cristóbal (Wiedenfeld 2006). An extensive survey in 1998 failed to find the subspecies (Vargas and Bensted-Smith 2000). | LC / EX |
| **Formicariidae** | Antioquia Brown-banded Antpitta *Grallaria milleri gilesi* | Antioquia, Colombia | 1939 (1878–2009) | This subspecies was recently described by Salaman et al. (2009) and has not been observed since its collection in 1878. It was treated as Critically Endangered in its description, but a number of potential sites were surveyed soon afterwards with no success and it is now thought probably extinct (P. Salaman *in litt.* 2011). Because it is poorly known there is a chance it may persist elsewhere, and so it is considered Critically Endangered (Possibly Extinct). | EN / CR(PE) |
| **Maluridae** | Macdonnell Ranges Grasswren *Amytornis textilis modestus* | Northern Territory, Australia | 1936 | The subspecies is treated here *sensu strictocontra* Dickinson (2003) and following Black (2010), who suggests splitting the species into two polytypic species *textilis* and *modestus*. The last record was a clutch of eggs taken in 1936 (Parker 1972). | LC / EX |
|  | Namoi Grasswren *Amytornis textilis inexpectatus* | C New South Wales, Australia | 1886 | This subspecies is recognised here *contra* Dickinson (2003) and following Black (2011b), who considers it a race of the putative species *A. modestus*. It was last recorded in 1886 (McAllan 1987). | LC / EX |
|  | Large-tailed Grasswren *Amytornis textilis macrourus* | S Western Australia | 1910 | This subspecies is recognised here *contra* Dickinson (2003) and following Black (2011a). Carter was last to collect it, in September 1910. It is now presumed extinct (Black 2011a). | LC / EX |
| **Meliphagidae** | Chatham Bellbird *Anthornis melanocephala* | Chatham Is, New Zealand | 1922 (1906–1938) | It was last recorded in 1906 on Little Mangere. An expedition in 1938 hoping to rediscover the species found no trace of it (Tennyson and Martinson 2006). | EX |
| **Dasyornithidae** | Western Rufous Bristlebird *Dasyornis broadbenti litoralis* | SW Australia | 1959 (1940–1977) | The last specimens were collected in 1906 and 1908 (Whittell 1936, Serventy and Whittell 1967), with the last credible sighting in 1940 (Glauert 1944). More recent surveys, beginning in 1977, have not found it (Blakers et al. 1984, Barrett et al. 2003). Unconfirmed reports from 1977 and 1980 are not considered to be accurate. | LC / EX |
| **Acanthizidae** | Lord Howe Gerygone *Gerygone insularis* | Lord Howe I, Australia | 1932 (1928–1936) | Abundant in 1928, but the species could not be found on a visit in 1936 and has not been seen since (Hindwood 1940). | EX |
| **Callaetidae** | South Island Kokako *Callaeas cinereus cinereus* | South I, New Zealand | 1967 | The taxon was last recorded in 1967 (Clout and Hay 1981). | EN / EX |
|  | Huia *Heteralocha acutirostris* | North I, New Zealand | 1907 | The last confirmed record dates from 1907 (BirdLife International 2011). | EX |
| **Cinclosomatidae** | Mount Lofty Ranges Quail-thrush *Cinclosoma punctatum anachoreta* | Mt Lofty Ranges, Australia | 1984 (1983–1984) | Birds were seen near Mt Bold in 1983, but a survey in 1984 failed to find the subspecies (Paton et al. 1994), so extinction was probably around that time. Further searches would be needed to confirm the extinction and it is best treated as Critically Endangered (Possibly Extinct). | LC / CR(PE) |
| **Cracticidae** | Western Pied Currawong *Strepera graculina ashbyi* | W Victoria, Australia | 1927 | Introgression from the neighbouring subspecies was probably initiated by habitat change in the 1830s (Garnett et al. 2011). The last time the subspecies can be certainly considered distinct was 1927 (Ashby 1927, Garnett et al. 2011). | LC / EX |
| **Campephagidae** | Cebu Bar-bellied Cuckooshrike *Coracina striata cebuensis* | Cebu, Philippines | 1927 (1906–1947) | The taxon has not been found since McGregor collected it in 1906, including a survey beginning in 1947 (Rabor 1959) and searches in the early 2000s (Paguntalan and Jakosalem 2008). | LC / EX |
|  | Cebu Blackish Cuckooshrike *Coracina coerulescens altera* | Cebu, Philippines | 1927 (1906–1947) | The taxon has not been found since McGregor collected it in 1906, including a survey beginning in 1947 (Rabor 1959) and searches in the early 2000s (Paguntalan and Jakosalem 2008). | LC / EX |
|  | Norfolk Island Long-tailed Triller *Lalage leucopyga leucopyga* | Norfolk Island | 1956 (1942–1969) | The taxon was apparently abundant in 1941, but has not been seen since 1942 (Schodde et al. 1983). The first comprehensive survey of Norfolk Island's birds in 1969 found none (Smithers and Disney 1969). A range of surveys since have also found no evidence (Schodde et al. 1983, Robinson 1988, Bell 1990). | LC / EX |
| **Oriolidae** | Cebu Dark-throated Oriole *Oriolus steerii assimilis* | Cebu, Philippines | 1954 (1906–2001) | The taxon was last recorded in 1906 (Walther and Jones 2008). It was not found in 2001(Paguntalan and Jakosalem 2008), but there were unconfirmed reports from around that time (P. G. Jakosalem *in litt.* 2011). These reports suggest a slight possibility of survival, so it is considered Critically Endangered (Possibly Extinct). | LC / CR(PE) |
|  | North Island Piopio *Turnagra tanagra* | North I, New Zealand | 1970 (1902–1970) | The genus *Turnagra* is included in Oriolidae following Johansson et al. (2011). The last substantiated record of this species was a specimen shot in 1902. There have been unconfirmed reports since, but none since around 1970 (Tennyson and Martinson 2006). | EX |
|  | Southern Piopio *Turnagra capensis capensis* | South I, New Zealand | 1905 | The taxon was last recorded in 1905, when it was said to be ‘fast expiring’. There have been only dubious sightings since (Tennyson and Martinson 2006). | EX / EX |
|  | Stephens Island Piopio *Turnagra capensis turnagra* | Stephens I, New Zealand | 1897 | The last record of the subspecies appears to be from 1897 (BirdLife International 2011). | EX / EX |
| **Rhipiduridae** | Lord Howe Fantail *Rhipidura fuliginosa cervina* | Lord Howe I, Australia | 1924 (1924–1928) | The subspecies was practically extinct in 1924 (Hindwood 1940) and was not found in a 1928 survey (Sharland 1929), or in any subsequent work on Lord Howe Island (Disney and Smithers 1972, Recher and Clark 1974). | LC / EX |
|  | Guam Rufous Fantail *Rhipidura rufifrons urianiae* | Guam | 1984 | The taxon used to be widespread, with its distinctive song frequently heard, but it has not been recorded since 1984 and is presumed extinct (Boles 2006). | LC / EX |
| **Monarchidae** | Negros Celestial Monarch *Hypothymis coelestis rabori* | Visayas, Philippines | 1974 (1959–1989) | The taxon was present on Negros in 1959, but is likely to be extinct there now (BirdLife International 2011). It was also not found during surveys on Sibuyan in 1989–1992 (Clement et al. 2006). Further surveys on Negros would be required to confirm its extinction, so it is considered Critically Endangered (Possibly Extinct). | VU / CR(PE) |
|  | Maupiti Monarch *Pomarea pomarea* | Maupiti, French Polynesia | 1823 | The species presumably became extinct soon after its type was collected in 1823 (BirdLife International 2011). | EX |
|  | Eiao Monarch *Pomarea fluxa* | Eiao, French Polynesia | 1982 (1977–1987) | The species was last recorded in 1977, and 10 years later a survey failed to find it (Thibault and Meyer 2001). It has not been seen since. | EX |
|  | Hiva Oa Monarch *Pomarea mendozae mendozae* | Hiva Oa, French Polynesia | 1983 (1975–1990) | Only one individual was seen despite many weekly searches in 1975. Expeditions in 1990, 1996 and 2000 found no birds. There were two reports in the late 1990s, but these are considered erroneous (Thibault and Meyer 2001). | EN / EX |
|  | Nuku Hiva Monarch *Pomarea nukuhivae* | Nuku Hiva, French Polynesia | 1954 (1930–1972) | It was last recorded in the 1930s and has not been seen in several surveys since, the first being in 1972 (Thibault and Meyer 2001). | EX |
| **Monarchidae** *cont.* | Ua Pou Monarch *Pomarea mira* | Marquesas, French Polynesia | 1987 (1985–1989) | The last confirmed record dates from 1985, and searches in 1989, 1990 and 1998 failed to find the species (Thibault and Meyer 2001). However, a possible report from 2010 has raised hopes that the species may still survive, with follow-up searches planned for 2012 (P. Raust *in litt*. 2011). In light of this, although the species was considered Extinct on the 2011 IUCN Red List (BirdLife International 2011), we treat it as Critically Endangered (Possibly Extinct) here. | CR(PE) |
|  | Guam Flycatcher *Myiagra freycineti* | Guam | 1983 | Common in forest until the 1970s, this species plummeted to extinction in 1983 (BirdLife International 2011). | EX |
| **Corvidae** | Hawaiian Crow *Corvus hawaiiensis* | Hawaiian Is, USA | 2003 (2002–2003) | The last two wild individuals were recorded in 2002 but did not return to their breeding site in 2003 (BirdLife International 2011). | EW |
| **Petroicidae** | Tiwi Hooded Robin *Melanodryas cucullata melvillensis* | Tiwi Is, Australia | 1994 (1992–1996) | The only records since its description in 1911 were at single sites on Melville and Bathurst Islands in 1991 and 1992 (Garnett et al. 2011). A thorough two-week survey in 1996 (Mason and Schodde 1997), as well as exhaustive subsequent work involving 400 man-days of searching (Woinarski et al. 2003), failed to find it. Despite the fairly recent latest record, it is considered extinct due to the extent of these searches. | LC / EX |
| **Mohoidae** | Kauai Oo *Moho braccatus* | Hawaiian Is, USA | 1993 (1987–1998) | The last report, of vocalisations, came from 1987. Surveys since have been unsuccessful and it was considered extinct by 1998 (BirdLife International 2011). | EX |
|  | Oahu Oo *Moho apicalis* | Hawaiian Is, USA | 1864 (1837–1890) | The species has not been recorded since 1837. None of the collectors visiting O‘ahu between 1890 and 1900 found any trace of it (Greenway 1967). | EX |
|  | Bishop's Oo *Moho bishopi* | Hawaiian Is, USA | 1991 (1981–2001) | It was last recorded in 1981 and considered extinct by 2001 (BirdLife International 2011). | EX |
|  | Hawaii Oo *Moho nobilis* | Hawaiian Is, USA | 1934 | The species was last seen in 1934 on Lana‘i (BirdLife International 2011). | EX |
|  | Kioea *Chaetoptila angustipluma* | Hawaiian Is, USA | 1859 | The last specimen was collected in 1859 (BirdLife International 2011). | EX |
| **Paridae** | Daito Varied Tit *Parus varius orii* | Daito-jima, Japan | 1961 (1938–1984) | It was last seen in 1938. Surveys of its habitat in 1984 and 1986, as well as more recent searches, found none (Gosler and Clement 2007). | LC / EX |
| **Hirundinidae** | Jamaican Golden Swallow *Tachycineta euchrysea euchrysea* | Jamaica | 1989 | The taxon has not been recorded since 1989 when it was seen in the Blue Mountains IBA. It is likely to be extinct (D.C. Wege *in litt.* 2011), but targeted surveys would be required to confirm this and it is best treated as Critically Endangered (Possibly Extinct). | VU / CR(PE) |
| **Cisticolidae** | Northern White-winged Apalis *Apalis chariessa chariessa* | Tana Valley, Kenya | 1978 (1961–1994) | The taxon has not been observed since 1961 (Ryan et al. 2006). Surveys since then, including in 1994, have found no evidence and it is likely to be extinct. However, these searches have not been specific (Butynski 1994); until targeted surveys have confirmed its absence it is best considered Critically Endangered (Possibly Extinct). | VU / CR(PE) |
| **Sylviidae** | Chatham Fernbird *Bowdleria rufescens* | Chatham Is, New Zealand | 1892 | The last record was from around 1892, when the species is thought to have become extinct (Tennyson and Martinson 2006). | EX |
|  | Aldabra Warbler *Nesillas aldabrana* | Aldabra, Seychelles | 1985 (1983–1986) | The last records were in 1983; intensive searches in 1986 confirmed that the species was extinct (BirdLife International 2011). | EX |
| **Sylviidae** *cont.* | Astrolabe Nightingale Reed-warbler *Acrocephalus luscinius astrolabii* | Yap, Micronesia | 1839 | The two type specimens were collected during Second Antarctic Expedition 1838–1839 (Kennerley and Pearson 2010). There do not appear to have been any further records of the subspecies. | CR / EX |
|  | Pagan Nightingale Reed-warbler *Acrocephalus luscinius yamashinae* | Pagan, Northern Mariana Islands | 1969 (1960–1978) | Became extinct on Pagan, to which it was confined, before 1981 (Bairlein et al. 2006). Local people stated that it persisted until the 1960s, but intensive searches in the late 1970s and early 1980s confirmed its extinction (Tenorio et al. 1979, Reichel et al. 1992). | CR / EX |
|  | Laysan Millerbird *Acrocephalus familiaris familiaris* | NW Hawaiian Is, USA | 1920 (1916–1923) | The taxon became extinct between 1916 and 1923 (BirdLife International 2011). | CR / EX |
|  | Moorea Reed-warbler *Acrocephalus caffer longirostris* | Moorea, French Polynesia | 1984 (1981–1986) | The last bird was seen in 1981 (Holyoak and Thibault 1984). Research in 1986–1987 found no evidence, but there was a possible sighting in 2003 (Cibois et al. 2008) and an unconfirmed record at Cook Bay in 2010 (A. Gouni *in litt.* 2011). In light of these reports it is considered Critically Endangered (Possibly Extinct). | EN / CR(PE) |
|  | Raiatea Reed-warbler *Acrocephalus caffer musae* | Raiatea, French Polynesia | 1896 (1870–1922) | This subspecies is recognised *contra* Dickinson (2003), following Cibois et al. (2008). It was collected by Garrett in the 1870s (Cibois et al. 2008), but the Whitney Expedition found none in 1922 (Kennerley and Pearson 2010). | EN / EX |
|  | Huahine Reed-warbler *Acrocephalus caffer garretti* | Huahine, French Polynesia | 1896 (1870–1921) | A few birds were collected by Garrett between 1870 and 1887. The Whitney Expedition in 1921 found none (Cibois et al. 2008). | EN / EX |
|  | Eastern Canary Islands Chiffchaff *Phylloscopus canariensis exsul* | Canary Is, Spain | 2006 (1950–2006) | There have been no recent records and the taxon is likely to be extinct (Bairlein et al. 2006). The date of its last record is unclear. As it is poorly known there is a chance it may survive, and so it is considered Critically Endangered (Possibly Extinct). | LC / CR(PE) |
|  | Fayyum Sardinian Warbler *Sylvia melanocephala norrisae* | Fayyum area, Egypt | 1939 | The taxon was common in the 1920s, but last seen in 1939. It is now considered Extinct (Bairlein et al. 2006). | LC / EX |
| **Zosteropidae** | Seychelles Yellow White-eye *Zosterops mayottensis semiflavus* | Marianne, Seychelles | 1900 (1892–1940) | Abbott took specimens of the subspecies in 1892 (Ridgway 1895). It probably went extinct around 1900, and certainly by 1940 (Penny 1974). | LC / EX |
|  | Guam Bridled White-eye *Zosterops conspicillatus conspicillatus* | Guam | 1983 | The most recent sighting was in June 1983 at Pajon Basin (Van Balen 2008). There is no reasonable doubt that it is now extinct (G. C. L. Dutson *in litt.* 2011). | EN / EX |
|  | Robust White-eye *Zosterops strenuus* | Lord Howe I, Australia | 1918 (1908–1928) | The species was common in 1908 (Hull 1909), but could not be found in 1928 (Sharland 1929). | EX |
|  | White-chested White-eye *Zosterops albogularis* | Norfolk Island | 1995 (1979–2010) | It has not been certainly recorded since 1979. Formal searches have failed to find any over the last two decades, although there have been scattered reports throughout this time (BirdLife International 2011). Despite being considered Critically Endangered currently by BirdLife International (2011), it is treated as Critically Endangered (Possibly Extinct) here on the basis of unsuccessful surveys in 2010 (G. C. L. Dutson *in litt.* 2011). | CR(PE) |
|  | Mukojima White-eye *Apalopteron familiare familiare* | N Bonin Is, Japan | 1936 (1930–1941) | The last specimen was taken in 1930 on Mukojima, by which time it was already extinct on Nakôdo-jima. It was not observed on Mukojima in a 1941 survey, and is probably now extinct (Van Balen 2008). Targeted searches would be required to definitely confirm this so it is currently treated as Critically Endangered (Possibly Extinct). | VU / CR(PE) |
| **Regulidae** | Guadalupe Ruby-crowned Kinglet *Regulus calendula obscurus* | Guadalupe, Mexico | 1978 (1953–2003) | The taxon is considered Extinct having not been observed since 1953, including a search in 2003 (Barton et al. 2004). | LC / EX |
| **Troglodytidae** | San Benedicto Rock Wren *Salpinctes obsoletus exsul* | San Benedicto, Mexico | 1952 (1903–1953) | Its extinction was caused by a volcanic eruption in 1952, with no birds found in a 1953 survey (Kroodsma and Brewer 2005). The lack of published records between 1903 and 1952 is presumably due to the low survey effort. | LC / EX |
|  | Guadalupe Bewick’s Wren *Thryomanes bewickii brevicaudus* | Guadalupe, Mexico | 1899 (1897–1901) | Recorded as ‘nearly extinct’ in 1897 (Kaeding 1905). Thorough searches around 1901 failed to find the taxon (Anthony 1901). | LC / EX |
|  | San Clemente Bewick's Wren *Thryomanes bewickii leucophrys* | San Clemente, USA | 1964 (1941–1986) | The last specimens from 1941, and the subspecies became extinct sometime prior to 1986 (Kroodsma and Brewer 2005). | LC / EX |
|  | Martinique Wren *Troglodytes aedon martinicensis* | Martinique | 1900 | The taxon has not been observed since around 1900 (Kroodsma and Brewer 2005). | LC / EX |
|  | Guadeloupe Wren *Troglodytes aedon guadeloupensis* | Guadeloupe | 1992 (1973–1992) | Although this taxon was believed extinct in 1914, small numbers were rediscovered in 1969 and 1973 (Kroodsma and Brewer 2005). However, there have been no subsequent records and the taxon is likely to have gone extinct (Levesque and Mathurin 2008). This cannot yet be confirmed without further searches, so it is best considered Critically Endangered (Possibly Extinct). | LC / CR(PE) |
| **Mimidae** | Barbados Scaly-breasted Thrasher *Margarops fuscus atlantica* | Barbados | 1987 (1987–1988) | The taxon was last recorded in 1987 and a survey of known localities in 1988 found none (Buden 1993). Follow up work in 1998 also failed to find the subspecies (Brewer 2001).However, a small amount of potential habitat remains inaccessible and unsurveyed (Buckley et al. 2009) and so the taxon could persist: it is best considered Critically Endangered (Possibly Extinct). | LC / CR(PE) |
| **Sturnidae** | Kosrae Starling *Aplonis corvina* | Kosrae, Micronesia | 1854 (1828–1880) | Known from two specimens collected in 1828, this species was extinct by the time Finsch visited the island in 1880 (BirdLife International 2011). | EX |
|  | Mysterious Starling *Aplonis mavornata* | Mauke, Cook Is | 1900 (1825–1975) | The species is known only from its type specimen, collected in 1825. The island was not visited by ornithologists until nearly 150 years later, by which time it had become extinct (BirdLife International 2011). | EX |
|  | Lord Howe Starling *Aplonis fusca hulliana* | Lord Howe I, Australia | 1923 (1918–1928) | The taxon has not been seen since 1918 (Hindwood 1940). A search in 1928 found none (Sharland 1929). | EX / EX |
|  | Norfolk Starling *Aplonis fusca fusca* | Norfolk Island | 1946 (1923–1968) | The taxon has not been seen since 1923 (Garnett 1992), but its absence was noted only in 1968 (Smithers and Disney 1969). | EX / EX |
|  | Rodrigues Starling *Necropsar rodericanus* | Rodrigues, Mauritius | 1744 (1726–1761) | It was reported by Tafforet in 1926, but has not been seen since. Pingré failed to find any in a 1961 visit (Cheke 1987). | EX |
|  | Reunion Starling *Fregilupus varius* | Réunion | 1855 (1850–1860) | The last specimen was shot in 1837, and the species became extinct between 1850 and 1860 (BirdLife International 2011). | EX |
| **Turdidae** | Bonin Thrush *Zoothera terrestris* | Ogasawara Is, Japan | 1859 (1828–1889) | Known from four specimens collected in 1828, this species could not be found when the island was next visited by an ornithologist, in 1889 (BirdLife International 2011). | EX |
|  | Kamao *Myadestes myadestinus* | Hawaiian Is, USA | 1990 (1985–1995) | The last confirmed sighting of the species was in 1985. Unconfirmed reports continued until 1991, but the lack of confirmed records despite intensive and targeted searches in 1995 and 1997, make it appropriate to classify this species as Extinct (BirdLife International 2011). | EX |
|  | Amaui *Myadestes woahensis* | Hawaiian Is, USA | 1825 | The species is known only from the type, collected by Bloxam from O‘ahu in 1825 (BirdLife International 2011). | EX |
| **Turdidae** *cont.* | Lanai Olomao *Myadestes lanaiensis lanaiensis* | Hawaiian Is, USA | 1933 | Last seen on Lana‘i in 1933 (Collar 2005), which is suggested as a likely extinction date by Clement and Hathway (2000). | CR(PE) / EX |
|  | Molokai Olomao *Myadestes lanaiensis rutha* | Hawaiian Is, USA | 1998 (1994–2001) | There were a few reports in the 1980s (Collar 2005) and it was last accurately recorded in 1994 (Clement and Hathway 2000). Most of its range was surveyed after without success, but Kamako‘u Peak and Oloku‘i have still not been explored (Reynolds and Snetsinger 2001). Until those sites have been surveyed, it is best considered Critically Endangered (Possibly Extinct). | CR(PE) / CR(PE) |
|  | Pines Solitaire *Myadestes elisabeth retrusus* | Isla de la Juventud, Cuba | 1935 | The taxon is thought to have died out in the 1930s, although one unconfirmed claim dates from the 1970s (Collar 2005). | NT / EX |
|  | Lord Howe Island Thrush *Turdus poliocephalus vinitinctus* | Lord Howe I, Australia | 1921 (1913–1928) | The subspecies was last recorded in 1913 and could not be found in 1928 (Sharland 1929, Hindwood 1940) | LC / EX |
|  | Norfolk Island Thrush *Turdus poliocephalus poliocephalus* | Norfolk Island | 1975 (1968–1976) | The taxon persisted until at least 1968, when two were mist-netted (Smithers and Disney 1969) and probably 1975, when one was seen (Schodde et al. 1983). Intensive searches since have proved fruitless (McKean et al. 1976, Garnett et al. 2011) and it is now considered Extinct. | LC / EX |
|  | Mare Island Thrush *Turdus poliocephalus mareensis* | Maré, New Caledonia | 1939 (1913–2003) | Saracin was last to collect it in 1913 (Warner 1947). It was regarded as near extinct in 1939. Surveys of Maré in the early 2000s did not find any evidence of the taxon, and it is considered Extinct (G. C. L. Dutson *in litt.* 2011). | LC / EX |
|  | Grand Cayman Thrush *Turdus ravidus* | Grand Cayman, Cayman Is | 1952 (1938–1965) | The last specimen was collected in 1911, with the last sight record in 1938. It was extinct by 1965 (Bradley 2000). | EX |
|  | Chinijo Chat *Saxicola dacotiae murielae* | Canary Is, Spain | 1935 (1913–1957) | Records from Allegranza and Montaña Clara have never been repeated, despite surveys in 1957, 1958 and 1971 (Bibby and Hill 1987). | EN / EX |
| **Cinclidae** | Cyprus Dipper *Cinclus cinclus olympicus* | Cyprus | 1945 | This taxon became extinct in 1945 (Flint and Stewart 1983). | LC / EX |
| **Estrildidae** | Southern Star Finch *Neochmia ruficauda ruficauda* | E Australia | 1996 (1995–1996) | Two birds were recorded in 1995 (Garnett et al. 2010). However, multiple recent targeted searches have failed to find any evidence of the taxon (Holmes 1996, 1998) and so it is considered Extinct. | NT / EX |
| **Fringillidae** | San Benito House Finch *Carpodacus mexicanus mcgregori* | San Benito, Mexico | 1945 (1940–1964) | The taxon was probably extinct by the 1940s and certainly by 1964 (Collar et al. 2010). | LC / EX |
|  | Bonin Grosbeak *Chaunoproctus ferreorostris* | Ogasawara Is, Japan | 1828 | It was last recorded in 1828. Local rumours that it persisted until the 1890s were never substantiated (Brazil 1991). | EX |
|  | Ou *Psittirostra psittacea* | Hawaiian Is, USA | 1989 | The species has not been recorded with certainty on Kaua‘i since 1989 nor on Big Island since 1987 (BirdLife International 2011). | CR(PE) |
|  | Lanai Hookbill *Dysmorodrepanis munroi* | Hawaiian Is, USA | 1918 | The species is known from only a single specimen collected in 1913, and from sightings in 1916 and 1918 (BirdLife International 2011). | EX |
|  | Lesser Koa-finch *Rhodacanthis flaviceps* | Hawaiian Is, USA | 1892 (1891–1893) | Specimens were collected on Big Island in 1891, but Perkins did not find the bird in 1893 and it has not been seen since (Greenway 1967). | EX |
|  | Greater Koa-finch *Rhodacanthis palmeri* | Hawaiian Is, USA | 1901 (1896–1906) | The last specimens were taken onBig Island in 1896, and ten years later it could not be found by Henshaw (BirdLife International 2011). | EX |
| **Fringillidae** *cont.* | Kona Grosbeak *Chloridops kona* | Hawaiian Is, USA | 1894 | The species was last collected in 1894 on Lana‘i (BirdLife International 2011). | EX |
|  | Greater Amakihi *Hemignathus sagittirostris* | Hawaiian Is, USA | 1901 | The specoes was last recorded in 1901 on Big Island (BirdLife International 2011). | EX |
|  | Lesser Akialoa *Hemignathus obscurus* | Hawaiian Is, USA | 1940 | The last report was in 1940 on Big Island (BirdLife International 2011). | EX |
|  | Maui Nui Akialoa *Hemignathus ellisianus lanaiensis* | Hawaiian Is, USA | 1892 | The taxon is lnown from only three specimens, collected in 1892 on Lana‘i (Olson and James 1995). | EX / EX |
|  | Oahu Akialoa *Hemignathus ellisianus ellisianus* | Hawaiian Is, USA | 1868 (1837–1900) | Known from only two specimens collected in 1837 (Olson and James 1995) and undocumented reports in 1937 and 1940 (Greenway 1967). These reports are not generally considered accurate and the taxon likely died out in the 1800s (E. VanderWerf *in litt.* 2011). | EX / EX |
|  | Kauai Akialoa *Hemignathus ellisianus stejnegeri* | Hawaiian Is, USA | 1969 | The taxon was last recorded in 1969 (Olson and James 1995), around which time it is thought to have gone extinct (E. VanderWerf *in litt.* 2011). Intensive surveys since have failed to find it (Gorresen et al. 2009). | EX / EX |
|  | Maui Nukupuu *Hemignathus lucidus affinis* | Hawaiian Is, USA | 1955 (1896–1994) | The taxon has not been certainly recorded since Perkins collected it in 1896 (Munro 1960). Research in 1994–1996 only yielded one possible sighting (Reynolds and Snetsinger 2001). Pratt (2010) suggests a few recent reports seem credible, but Pratt and Pyle (2000) are sceptical of all 20^th^ century records. | CR(PE) / CR(PE) |
|  | Oahu Nukupuu *Hemignathus lucidus lucidus* | Hawaiian Is, USA | 1880 (1860–1900) | Perkins found evidence that many existed in the forests of Oahu in 1860, but no collectors found any trace of it in the 1890s (Munro 1960). | CR(PE) / EX |
|  | Kauai Nukupuu *Hemignathus lucidus hanapepe* | Hawaiian Is, USA | 1950 (1899–1999) | Two were taken in 1899 by Munro (1960), who doubts it has been seen since. There were unconfirmed reports in 1960 and 2007 (Pratt 2010). Intensive searches in the 1990s were unsuccessful (Pratt and Pyle 2000). | CR(PE) / CR(PE) |
|  | Lanai Alauahio *Paroreomyza montana montana* | Hawaiian Is, USA | 1949 (1937–1960) | It was last recorded in 1937 and considered extinct by 1960 (Munro 1960). | EN / EX |
|  | Kakawahie *Paroreomyza flammea* | Hawaiian Is, USA | 1970 (1961–1979) | The last sightings were between 1961 and 1963 on Moloka‘i. The Hawaiian Forest Bird Survey beginning in 1979 failed to find the species, and there have been no records since (Gorresen et al. 2009). | EX |
|  | Oahu Alauahio *Paroreomyza maculata* | Hawaiian Is, USA | 1985 | The last well-documented observation of this species was in 1985, with a reasonably convincing, but unconfirmed, record in 1990 (BirdLife International 2011). | CR(PE) |
|  | Maui Akepa *Loxops coccineus ochraceus* | Hawaiian Is, USA | 1994 (1988–2000) | Last reported in 1988. Searches in the 1990s were inconclusive, with possible audio evidence obtained (Pratt 2010), amd in light of this it is considered Critically Endangered (Possibly Extinct). | EN / CR(PE) |
|  | Oahu Akepa *Loxops coccineus wolstenholmei* | Hawaiian Is, USA | 1968 (1930–2005) | The last convincing report dates from 1930 (Donaghho 1963). It is considered extinct by Pratt (2005). | EN / EX |
|  | Ula-ai-hawane *Ciridops anna* | Hawaiian Is, USA | 1892 | It has not been found since a specimen was collected in 1892 on Big Island (Greenway 1967). There is a possible but unlikely record from 1937 (Munro 1960). | EX |
|  | Hawaii Mamo *Drepanis pacifica* | Hawaiian Is, USA | 1898 | The species was last recorded in 1898 (BirdLife International 2011). | EX |
| **Fringillidae** *cont.* | Black Mamo *Drepanis funerea* | Hawaiian Is, USA | 1928 (1907–1949) | It was last collected in 1907 on Lana‘i. Searches since then failed to find it, and it was considered extinct by 1949 (BirdLife International 2011). | EX |
|  | Laysan Honeycreeper *Himatione sanguinea freethii* | Hawaiian Is, USA | 1923 | Three were seen by Wetmore when he arrived on Laysan in 1923 (Munro 1960). This is widely accepted as the date of extinction, as the birds were not seen following a sandstorm later that year, and have never been seen since (E. VanderWerf*in litt.* 2011). | LC / EX |
|  | Poo-uli *Melamprosops phaeosoma* | Hawaiian Is, USA | 2004 | One of the last three known individuals in the wild was taken into care in September 2004, but it died in November 2004. The other two birds hadnot been seen on Maui since December 2003 and February 2004 respectively (BirdLife International 2011). | CR(PE) |
| **Parulidae** | Bachman's Warbler *Vermivora bachmanii* | S USA | 1967 (1958–1975) | The last confirmed records came from near Charleston, South Carolina in 1958-1961. Specific searches in 1975-1979 failed to find the species (Hamel 2011), but there have been unconfirmed reports since and it is best treated as Critically Endangered (Possibly Extinct) until its entire potential habitat has been surveyed (BirdLife International 2011). | CR(PE) |
| **Icteridae** | Grand Cayman Oriole *Icterus leucopteryx bairdi* | Grand Cayman, Cayman Islands | 1967 | This taxon was last seen in 1967 and has since been extirpated (Bradley 2000). | LC / EX |
|  | Slender-billed Grackle *Quiscalus palustris* | Rio Lerma, Mexico | 1910 | The species was last recorded in 1910, and presumably became extinct soon after (BirdLife International 2011). | EX |
| **Emberizidae** | Santa Barbara Song Sparrow *Melospiza melodia graminea* | Santa Barbara I, USA | 1965 (1960–1970) | The taxon became extinct in the 1960s (Fuller 2000). | LC / EX |
|  | Dusky Seaside Sparrow *Ammodramus maritimus nigrescens* | SE USA | 1980 | The last wild record was in 1980 (Walters 1992). | LC / EX |
|  | Todos Santos Rufous-sided Sparrow *Aimophila ruficeps sanctorum* | Todos Santos Is, Mexico | 1953 (1928–1977) | The taxon was once a common breeder on the islands (Grinnell 1928), but was not found during several visits in the late 1970s (Wilbur 1987) and does not appear to have been seen since, so it has probably gone extinct. Further surveys are required to confirm this however, and the taxon is best considered Critically Endangered (Possibly Extinct). | LC / CR(PE) |
|  | Hooded Seedeater *Sporophila melanops* | Brazil | 1916 (1823–2008) | The species is only known from the type, which was collected in 1823 on the banks of Rio Araguaia. Searches of the river's floodplain in 2008 and 2009 found no evidence of the species, but more comprehensive surveys would be required to confirm its extinction and so it is best considered Critically Endangered (Possibly Extinct) (BirdLife International 2011). | CR(PE) |
|  | St Kitts Bullfinch *Loxigilla portoricensis grandis* | St Kitts | 1880 | The taxon was common in the forests of Mt Misery in 1880 (Bond 1956), but has since gone extinct. | LC / EX |
|  | Guadalupe Spotted Towhee *Pipilo maculatus consobrinus* | Guadalupe, Mexico | 1897 | It was found by Palmer in 1875 (Thayer and Bangs 1908), but not by an expedition in 1897 or in any subsequent work (Kaeding 1905). | LC / EX |
| **Thraupidae** | Samana Eastern Chat Tanager *Calyptophilus frugivorus frugivorus* | Samaná, Dominican Republic | 1987 (1982–1992) | The taxon has not been reliably recorded since 1982 (Raffaele et al. 1998). Concerted efforts to find it have been unsuccessful (Dod 1992) and the subspecies is believed to be extinct (Keith et al. 2003). | VU / EX |
| **Thraupidae** *cont.* | Gonave Eastern Chat Tanager *Calyptophilus frugivorus abbotti* | Gonâve I, Haiti | 1991 (1977–2006) | Last recorded in 1977, and was not recorded in recent searches (Latta et al. 2006). However, Gonâve is not thought to have been comprehensively surveyed (D.C. Wege *in litt.* 2011) and until it has been the taxon is best considered Critically Endangered (Possibly Extinct). | VU / CR(PE) |

^1^Minimum date = date of last record. Maximum date = date of the first unsuccessful survey of the taxon’s range. The estimated extinction dateis taken as the mid-point of the minimum and maximum (where available)

^2^Where BirdLife International (2011) is listed, see this source for primary references

^3^Species status = 2011 IUCN Red List category; subspecies status = our assessment, where EX = Extinct, EW = Extinct in the Wild, and CR(PE) = Critically Endangered (Possibly Extinct)
