# Supplementary material for: Global Patterns and Drivers of Avian Extinctions at the Species and Subspecies Level
Source: PLoS One. 2012 Oct 8;7(10):e47080. doi: 10.1371/journal.pone.0047080 (PMC3466226; doi:10.1371/journal.pone.0047080)
Supplement: Table S2 — Avian taxa considered extinct and recognised taxonomically by other authors but not by BirdLife International (2011) for species or Dickinson (2003) for subspecies. Asterisks indicate four subspecies that are recognised by Dickinson (2003) but were excluded in this study, with explanations given in the notes. (DOCX) [file pone.0047080.s002.docx]

| Taxon | Notes |
| --- | --- |
| North Island Kiwi *Apteryx owenii iredalei* | Noted by Dickinson (2003) as not being distinct. |
| West Coast Spotted Kiwi *Apteryx australis occidentalis* |  |
| Key West Bobwhite *Colinus virginianus insulanus* * | Not recognised following Aldrich (1946) who considered it synonymous with *C. v. floridianus*. Apparently overlooked by Dickinson (2003). |
| Turkish Black Francolin *Francolinus francolinus billypayni* | Not considered distinct from *F. f. francolinus* by Dickinson (2003). |
| Double-banded Argus *Argusianus bipunctatus* | Not recognised by BirdLife International following Davison and McGowan (2009). |
| Rennell Island Teal *Anas gracilis remissa* | Noted by Dickinson (2003) as not being distinct. |
| Tristram’s Pintail*Anas acuta modesta* |  |
| Mariana Mallard *Anas oustaleti* | Considered to be a hybrid between Mallard *A. platyrhynchos* and Pacific Black Duck *A. superciliosa* by BirdLife International (2011). |
| Levant Darter*Anhinga rufa chantrei* |  |
| Sharpe’s Rail *Gallirallus sharpei* | Considered most likely to have been a colour morph of Buff-banded Rail *G. philippensis* (BirdLife International 2011). |
| Intact Rail *Gymnocrex plumbeiventris intactus* |  |
| Iwo-jima Rail *Porzana cinerea brevipes* | Noted by Dickinson (2003) as not being distinct. |
| Luzon Sarus Crane *Grus antigone luzonica* | Not considered distinct from *G. a. sharpei* by Dickinson (2003). |
| Ebon Island Fruit-dove *Ptilinopus porphyraceus marshallianus* |  |
| Aruba Amazon *Amazona barbadensis canifrons* |  |
| Miyako Island Kingfisher *Todiramphus cinnamominus miyakoensis** | Only known from the type specimen, which is now thought to be from Guam, where the nominate form was found, rather than Miyako, and hence*miyakoensis* is likely invalid (D. Allen *in litt.* 2011). |
| North Island Stitchbird *Notiomystis cincta cincta* * | Not recognised following Higgins and Christidis (2009) who describe overlap in characters. |
| Meinertzhagen’s Warbler *Sylvia deserticola ticehursti* * | Recent evidence indicates it is not distinct from the extant *S. d. maroccana* (Bairlein et al. 2006). |
| Daito Winter Wren *Troglodytes troglodytes orii* |  |
| Yakushima Seven Island Thrush *Turdus celaenops yakushimensis* |  |
| Darwin’s Large Ground-finch *Geospiza magnirostris magnirostris* |  |

Table S2. Avian taxa considered extinct and recognised taxonomically by other authors but not by BirdLife International (2011) for species or Dickinson (2003) for subspecies. Asterisks indicate four subspecies that are recognised by Dickinson (2003) but were excluded in this study, with explanations given in the notes (Supplementary Table 3). e been considered extinct by other authors, but are regarded as extant listed in Supplementary Table
