# Supplementary material for: Global Patterns and Drivers of Avian Extinctions at the Species and Subspecies Level
Source: PLoS One. 2012 Oct 8;7(10):e47080. doi: 10.1371/journal.pone.0047080 (PMC3466226; doi:10.1371/journal.pone.0047080)
Supplement: Table S3 — Avian taxa that have been considered extinct by other authors but are considered extant by this study, either because they have been recently recorded/rediscovered, or because there is insufficient evidence to presume extinction or possible extinction, despite a lack of recent records (see Notes for justifications). (DOCX) [file pone.0047080.s003.docx]

Table S3.Avian taxa that have been considered extinct by other authors but are considered extant by this study, either because they have been recently recorded/rediscovered, or because there is insufficient evidence to presume extinction or possible extinction, despite a lack of recent records (see notes for justifications)

| Family | Taxon | Distribution | Source^1^ | Notes^2^ |
| --- | --- | --- | --- | --- |
| **Tinamidae** | Magdalena Tinamou *Crypturellus erythropus saltuarius* | Magdalena Valley, Colombia | Hume and Walters (2012) | Recently, a number of unconfirmed reports and the discovery of remnant habitat (Donegan et al. 2003a, b) suggest it is quite likely to be extant. |
| **Cracidae** | Natterer’s Curassow *Crax fasciolata pinima* | NE Brazil | Dickinson (2003) | Considered extant by del Hoyo (1994) and other authors. |
| **Phasianidae** | Himalayan Quail *Ophrysia superciliosa* | Uttaranchal, India | Hume and Walters (2012) | Probably extant: it is very difficult to detect, and there are possible sightings from 2003 and more recent local reports (Butchart et al. 2005, BirdLife International 2011). |
| **Anatidae** | Crested Shelduck *Tadorna cristata* | NE Asia | Hume and Walters (2012) | Unrecorded since 1964, but large areas of its range have not been adequately surveyed. There are recent claims from China and the species may well survive (Butchart et al. 2005, BirdLife International 2011). |
|  | Pink-headed Duck *Rhodonessa caryophyllacea* | SE Asia | Hume and Walters (2012) | Although not recorded since 1949, there were credible local reports of the species in 2006 from a wetland in northern Myanmar, and further surveys are needed (Butchart et al. 2005, BirdLife International 2011). |
| **Threskiornithidae** | Principe Olive Ibis *Bostrychia olivacea rothschildi* | Príncipe I, São Tomé and Príncipe | Dickinson (2003) | Although unrecorded between 1901 and 1971, there have been subsequent records including in 1991 and 1997 (Jones and Tye 2006). |
| **Accipitridae** | White-collared Kite *Leptodon forbesi* | NE Brazil | Hume and Walters (2012) | Confirmed extant, e.g. recorded at 12 sites in 2007 (BirdLife International 2011). |
|  | Car Nicobar Sparrowhawk *Accipiter butleri butleri* | Car Nicobar, India | Hume and Walters (2012) | Likely to still be extant, although it has been rarely seen: there have only been unconfirmed sightings in the last century but the island is poorly studied, and the species is ‘extremely shy’ (BirdLife International 2001). |
| **Rallidae** | Jamaican Black Rail *Laterallus jamaicensis jamaicensis* | Greater Antilles | Hume and Walters (2012) | Recently recorded in the Black River Morass Important Bird Area (D.C. Wege *in litt.* 2011). |
|  | New Caledonian Rail *Gallirallus lafresnayanus* | Grande Terre, New Caledonia | Hume and Walters (2012) | The lack of recent records is most likely because of inadequate field effort in inaccessible montane forests (BirdLife International 2011). |
|  | Flores Rail *Lewinia pectoralis exsul* | Flores, Indonesia | Hume and Walters (2012) | Only known from four specimens and last recorded in 1959, but there is no compelling reason why it should have gone extinct (Taylor and Van Perlo 1998). J. A. Eaton (*in litt.* 2011) suggests there is “no chance” of its extinction, and that the lack of records is attributable to minimal survey effort in its habitat. |
|  | Samoan Moorhen *Gallinula pacifica* | Savai‘i, Samoa | Hume and Walters (2012) | No definite recent records, but there have been unconfirmed reports. Extensive habitat remains and the species is likely to be shy and possibly nocturnal, making it difficult to detect (BirdLife International 2011). |
|  | Makira Moorhen *Gallinula silvestris* | Makira, Solomon Islands | Hume and Walters (2012) | There have been some credible recent reports, and searches to date have been inadequaste to presume it has gone extinct (BirdLife International 2011). |
| **Turnicidae** | Tawi-tawi Small Buttonquail *Turnix sylvaticus suluensis* | Sulu Archipelago, Philippines | Hume and Walters (2012) | No records since the 1950s, but this is likely to be due to the lack of fieldwork on Tawi-tawi and the species being a cryptic grassland specialist (BirdLife International 2011). |
| **Charadriidae** | Javan Lapwing *Vanellus macropterus* | Java, Indonesia | Hume and Walters (2012) | There is a lack of recent records but the species’s habitat has not been adequately surveyed (Butchart et al. 2005, BirdLife International 2011). |
| **Columbidae** | Ogasawara Wood-pigeon *Columba janthina nitens* | Ogasawara Is, Japan | Hume and Walters (2012), Dickinson (2003) | Previously thought to have gone extinct during the 1980s but there have been a number of recent reports, including photographs (D. Allen *in litt.* 2011). It is considered rare but extant, with a population of 30–40 birds (Brazil 2009). |
|  | Seychelles Turtle-dove *Nesoenas picturata rostrata* | Seychelles | Hume and Walters (2012) | The taxon has been swamped by introduced *N. p. picturata* over much of its range (Gibbs et al. 2001), but a few *rostrata*-types still survive on Aride, Cousin, Cousine and Bird (Skerrett and Disley 2011). |
|  | Catanduanes Bleeding-heart *Gallicolumba luzonica rubiventris* | Catanduanes I, Philippines | Hume and Walters (2012) | Much of this taxon’s forest habitat is left on Catanduanes (D. Allen *in litt.* 2011), and there have been recent reports (S. Wamelink *in litt.* 2008). |
|  | Sulu Bleeding-heart *Gallicolumba menagei* | Sulu Archipelago, Philippines | Hume and Walters (2012) | Thought to survive based on unconfirmed reports from multiple islands around 1995 (Butchart et al. 2005, BirdLife International 2011). |
|  | Solomon Islands Ground-dove *Gallicolumba jobiensis chalconota* | Solomon Islands | Hume and Walters (2012) | Only known from four specimens but considered likely to be extant (Dutson 2011). |
|  | Cebu Amethyst Brown-dove *Phapitreron amethystinus frontalis* | Cebu, Philippines | Dickinson (2003) | Confirmed extant, with multiple records since 2007 (P.G. Jakosalem *in litt.* 2011). |
|  | Negros Fruit-dove *Ptilinopus arcanus* | Negros, Philippines | Hume and Walters (2012) | No confirmed records since 1953, but it may have been observed in 2003. It could also conceivably occur on Panay, where searches are required (Butchart et al. 2005, BirdLife International 2011). |
| **Psittacidae** | Cebu Hanging-parrot *Loriculus philippensis chrysonotus* | Cebu, Philippines | Hume and Walters (2012), Dickinson (2003) | There have been a handful of unconfirmed sightings which need to be followed up, including a recent record in south Cebu (P.G. Jakosalem *in litt.* 2011). |
|  | Challenger Red-and-blue Lory *Eos histrio challengeri* | Miangas I, Indonesia | Hume and Walters (2012) | Nothing is known about its status (Collar 1997) and even the exact origin of the type specimens, from which it is exclusively known, is uncertain (N.J. Collar *in litt.* 2011). It is better treated as Data Deficient. |
|  | New Caledonian Lorikeet *Charmosyna diadema* | New Caledonia | Hume and Walters (2012) | Not reliably recorded for nearly 100 years, but *Charmosyna* lorikeets are notoriously difficult to detect and there have been unconfirmed reports during this time, so it is better treated as extant at present (BirdLife International 2011). |
|  | Glaucous Macaw *Anodorhynchus glaucus* | N Argentina | Hume and Walters (2012) | There have been few recent records but it is better considered extant because much of its formerly large range has not been adequately searched (Tobias et al. 2006, BirdLife International 2011). |
| **Strigidae** | Norfolk Island Boobook*Ninox novaeseelandiae undulata* | Norfolk Island | Hume and Walters (2012), Dickinson (2003) | The population was reduced to one female, but the subspecies is treated as extant because a hybrid population survives descended from this bird and an introduced male *N. n. novaeseelandiae* (Garnett et al. 2011). |
| **Apodidae** | Southern Vanuatu Swiftlet *Aerodramus spodiopygius ingens* | S Vanuatu | Hume and Walters (2012) | This taxon has recently been reported and is likely to remain extant (G.C.L. Dutson *in litt.* 2011). |
| **Trochilidae** | Coppery Thorntail *Discosura letitiae* | Bolivia | Hume and Walters (2012) | Only known from two specimens labelled as deriving from Bolivia, but this taxon could occur anywhere in the country, including the poorlyknown lowlands in the north, or even in parts of Brazil that used to belong to Bolivia. It is better considered Data Deficient (Tobias et al. 2006, BirdLife International 2011). |
|  | Bogota Sunangel *Heliangelus zusii* | Colombia | Hume and Walters (2012) | Known from a single specimen purchased in Bogotá in 1909. It could have come from anywhere in the country; some Bogotá specimens are even known to have been collected in Ecuador. It is considered Data Deficient (Tobias et al. 2006, BirdLife International 2011). |
| **Coraciidae** | Sri Lanka Broad-billed Roller *Eurystomus orientalis irisi* | Sri Lanka | Dickinson (2003) | There is no evidence that the subspecies is extinct, although the population has declined (De Zoysa and Raheem 1987, Fry 2001). |
| **Alcedinidae** | Malaita Variable Kingfisher *Ceyx lepidus malaitae* | Malaita, Solomon Islands | Hume and Walters (2012) | Seen frequently in 1997 (G.C.L. Dutson *in litt.* 2011). The lack of records since then is presumably just because of the access difficulties associated with Malaita. |
|  | Sakaraha Pygmy-kingfisher *Ceyx madagascariensis dilutus* | Sakaraha, Madagascar | Hume and Walters (2012) | The species still occurs, rarely, around the type locality of *dilutus*, though it has been suggested that *dilutus* may not be a valid subspecies (R. Safford *in litt.* 2011). |
|  | Javan Blue-banded Kingfisher *Alcedo euryzona euryzona* | Java, Indonesia | Hume and Walters (2012) | The taxon is unlikely to be extinct. It was mist-netted five times in 2000–2001 at Halimun National Park (Noske et al. 2011), an area which has not undergone much change since (B. van Balen *in litt.* 2011). |
| **Ramphastidae** | Northern White-mantled Barbet *Capito hypoleucos hypoleucos* | C Andes, Colombia | Hume and Walters (2012) | The taxon is confirmed as extant (T.M. Donegan *in litt.* 2011). |
|  | Botero White-mantled Barbet *Capito hypoleucos carrikeri* | Antioquia, Colombia | Hume and Walters (2012) | The taxon is confirmed as extant (T.M. Donegan *in litt.* 2011). |
| **Pittidae** | Bougainville Black-faced Pitta *Pitta anerythra pallida* | Bougainville, Papua New Guinea | Hume and Walters (2012) | There have been no recent records, but it may have been overlooked by observers, and it is likely to survive (G.C.L. Dutson *in litt.* 2011). |
|  | Choiseul Black-faced Pitta *Pitta anerythra nigrifrons* | Choiseul, Solomon Islands | Hume and Walters (2012) | There have been no recent records, but it may have been overlooked by observers, and it is likely to survive (G.C.L. Dutson *in litt*. 2011). |
| **Cotingidae** | Northern Purpletuft *Iodopleura pipra leucopygia* | NE Brazil | Dickinson (2003) | The subspecies was only considered extinct because of a mislabelling of specimens (Schuchmann 1999). |
| **Formicariidae** | Northern Giant Antpitta *Grallaria gigantea lehmanni* | Magdalena Valley, Colombia | Hume and Walters (2012) | The taxon was formerly rare, but plenty of habitat remains and it is unlikely to have gone extinct (T.M. Donegan *in litt.* 2011). |
|  | Ecuadorian Giant Antpitta *Grallaria gigantea hylodroma* | Ecuador | Hume and Walters (2012) | This taxon is confirmed to be extant, occurring at a number of sites, many of which are currently protected by landowners (Freile et al. 2010, J.F. Freile *in litt.* 2011). |
|  | Tachira Antpitta *Grallaria chthonia* | Táchira, Venezuela | Hume and Walters (2012) | The taxon may well persist near the type locality, where further searches are required (Tobias et al. 2006, BirdLife International 2011). |
| **Furnariidae** | Peruvian Scale-throated Earthcreeper *Upucerthia dumetaria peruana* | Puno region, Peru | Hume and Walters (2012) | This taxon is only known from two specimens collected over 50 years ago (Remsen 2003), but there are no obvious reasons why it may have gone extinct, and dedicated searches are required. |
| **Furnariidae** *cont.* | Northern Stripe-crowned Spinetail *Cranioleuca pyrrhophia rufipennis* | N Bolivian Andes | Hume and Walters (2012) | This taxon has not been recorded for at least 50 years, but it is considered unlikely to have gone extinct (Remsen 2003). |
| **Meliphagidae** | Eastern Yellow Chat *Epthianura crocea macgregori* | C Queensland, Australia | Dickinson (2003) | Confirmed extant (Houston et al. 2004) and is now considered Endangered (Garnett et al. 2011). |
| **Acanthizidae** | King Island Brown Thornbill *Acanthiza pusilla archibaldi* | King I, Australia | Dickinson (2003) | There have only been around a dozen records since its discovery, the latest in 2002, but as it has always been so rarely recorded it should not be considered extinct. The population is estimated at fewer than 50 birds (Garnett et al. 2011). |
| **Campephagidae** | Marinduque Blackish Cuckooshrike *Coracina coerulescens deschauenseei* | Marinduque, Philippines | Hume and Walters (2012) | There have been no recent records, but few ornithologists have been to Marinduque and there is still forest on the island where it is likely to survive (D. Allen *in litt.* 2011). |
| **Monarchidae** | Manua Shrikebill *Clytorhynchus vitiensis powelli* | Manu‘a Is, American Samoa | Hume and Walters (2012) | Considered extant on the islands of Manu‘a, Ofu and Olosega (Watling 2004), although it may have been extirpated from Tau (Clement et al. 2006). |
| **Remizidae** | Dusky Penduline-tit *Remiz pendulinus nigricans* | Iran | Dickinson (2003) | There is no evidence to suggest this subspecies has gone extinct, and the lack of records is more likely to be because of lack of searches. |
| **Hirundinidae** | White-eyed River-martin *Eurochelidon sirintarae* | C Thailand | Anon (2000) | This taxon’s breeding range is unknown, and further searches of possible habitat are needed, including in Myanmar (Butchart et al. 2005, BirdLife International 2011). |
| **Pycnonotidae** | Cebu Streak-breasted Bulbul *Ixos siquijorensis monticola* | Cebu, Philippines | Dickinson (2003) | Recently rediscovered, and found regularly in a recent survey of Cebu (Paguntalan and Jakosalem 2008). |
| **Sylviidae** | Vanua Levu Thicketbird *Trichocichla rufa clunei* | Vanua Levu, Fiji | Hume and Walters (2012) | This taxon has not been observed since the 1970s, but it is thought likely to be extant (G.C.L. Dutson*in litt.* 2011). |
|  | Babar Stubtail *Urosphena subulata advena* | Babar I, Indonesia | Hume and Walters (2012) | Confirmed extant and found to be common on Babar in 2009 and 2011 (C.R. Trainor *in litt.* 2011). |
|  | Nauru Reed-warbler *Acrocephalus rehsei* | Nauru | Dickinson (2003) | Confirmed extant and remains locally common on Nauru (BirdLife International 2011). |
|  | Daito Bush-warbler *Cettia diphone restricta* | Okinawa, Japan | Dickinson (2003) | Recent genetic analysis has shown that a resident population in Okinawa is of this taxon, formerly thought to have gone extinct (Manabu et al. 2002). |
|  | Western Turner’s Eremomela *Eremomela turneri kalindei* | E Africa | Hume and Walters (2012) | Unsurveyed forest persists in its known range and the taxon is likely to survive (L.D.C. Fishpool *in litt.* 2011). |
|  | Chapin’s Crombec *Sylvietta leucophrys chapini* | Lendu Plateau, DRC | Hume and Walters (2012) | Unsurveyed forest persists in its known range and the taxon is likely to survive (L.D.C. Fishpool *in litt.* 2011). |
| **Timaliidae** | Black-browed Babbler *Malacocincla perspicillata* | South Kalimantan, Indonesia | Hume and Walters (2012) | This taxon is only known from one specimen, of uncertain origin, but habitat remains within the putative distribution, and the taxon is better considered Data Deficient (Butchart et al. 2005, BirdLife International 2011). |
|  | Javan Large Wren-babbler *Napothera macrodactyla lepidopleura* | Java, Indonesia | Hume and Walters (2012) | This taxon is probably still extant (N.J. Collar *in litt.* 2011). It was recorded recently in Alas Purwo National Park (Grantham 2000). |
| **Timaliidae** *cont.* | Burmese Jerdon’s Babbler *Chrysomma altirostre altirostris* | Irrawaddy-Sittang Plain, Myanmar | Hume and Walters (2012), Dickinson (2003) | Although this taxon has not been seen since 1941 (Fuller 2000), adequate searches have not been carried out, and it probably survives(BirdLife International 2011, J.C. Eames *in litt.* 2011), with a possible sighting in 1994(T. Htin Hla *in litt.* 2011) |
|  | Amik Golu Bearded Parrotbill *Panurus biarmicus kosswigi* | Amik Gölü area | Dickinson (2003) | Although Lake Amik Gölü has been drained, this taxon persists in the surrounding area (Robson 2007). |
| **Mimidae** | Cozumel Thrasher *Toxostoma guttatum* | Cozumel I, Mexico | Hume and Walters (2012) | This taxon has only infrequently been seen since a devastating hurricane in 1988, with the last confirmed records during a survey in 2004 followed by claims from 2006. It would be premature to treat it as extinct (Tobias et al. 2006, BirdLife International 2011). |
| **Turdidae** | Kibale Black-eared Thrush *Zoothera cameronensis kibalensis* | Uganda | Hume and Walters (2012) | This taxon is considered likely to survive, given that suitable habitat remains (L.D.C. Fishpool *in litt.* 2011). |
|  | Choiseul Russet-tailed Thrush *Zoothera heinei choiseuli* | Choiseul, Solomon Islands | Hume and Walters (2012) | This taxon is only known from a single specimen collected in 1904 from the highest mountain on Choiseul, but the island is poorly known and the taxon probably survives (Dutson 2011). |
|  | Lifou Island Thrush *Turdus poliocephalus pritzbueri* | Tanna, Vanuatu | Hume and Walters (2012), Dickinson (2003) | This taxon has gone extinct on Lifou, but a population survives on Tanna (Collar 2005). |
|  | Cauca Black-hooded Thrush *Turdus olivater caucae* | Cauca, Colombia | Hume and Walters (2012) | The species is tolerant of habitat degradation and it is thought unlikely that this poorlyknown subspecies has gone extinct (T.M. Donegan *in litt.* 2011). |
| **Muscicapidae** | Rueck’s Blue-flycatcher *Cyornis ruckii* | Sumatra, Indonesia | Collen et al. (2010) | This taxon has not been recorded since 1918, but the Sumatran lowlands have not been adequately surveyed (Butchart et al. 2005, BirdLife International 2011). |
| **Dicaeidae** | Orange-breasted Flowerpecker *Dicaeum trigonostigma pallidius* | Cebu, Philippines | Dickinson (2003) | This taxon was rediscovered and found regularly during a recent survey of Cebu’s forests (Paguntalan and Jakosalem 2008). |
| **Parulidae** | New Providence Yellowthroat *Geothlypis rostrata rostrata* | New Providence, Bahamas | Hume and Walters (2012) | This taxon is known to have been rare in the 1990s (Raffaele et al. 1998), and there is no evidence that it has since gone extinct. |
|  | Semper’s Warbler *Leucopeza semperi* | St Lucia | Hume and Walters (2012) | There have been recent unconfirmed reports of this species, including in 2003, and its habitat has not been sufficiently surveyed for the bird to be considered extinct (Tobias et al. 2006, BirdLife International 2011). |
| **Emberizidae***.* | Antioquia Brush-finch *Atlapetes blancae* | Antioquia, Colombia | Hume and Walters (2012) | This species has not been reported at the type locality since 1971 despite searches in 2007 and 2008, but its range is so poorly known that it should not be considered extinct (BirdLife International 2011). |

^1^Refers to a recently published source in which the taxon is considered extinct

^2^The word ‘extinct’ is used as shorthand for ‘Extinct or Critically Endangered (Possibly Extinct)’ in this column
