# Supplementary material for: Global Patterns and Drivers of Avian Extinctions at the Species and Subspecies Level
Source: PLoS One. 2012 Oct 8;7(10):e47080. doi: 10.1371/journal.pone.0047080 (PMC3466226; doi:10.1371/journal.pone.0047080)
Supplement: Table S4 — The distribution of extinct and threatened taxa across avian families. L (lower) and U (upper) q values indicate the significance of differences from the expected values, i.e. fewer extinctions/lower degree of threat and more extinctions/higher degree of threat than expected, respectively. Q values equal zero when none of the simulated values crossed thresholds. Threatened species are those classified as such by BirdLife International (2011) in their IUCN Red List assessment. (DOCX) [file pone.0047080.s004.docx]

Table S4. The distribution of extinct and threatened taxa across avian families. L (lower) and U (upper) *q* values indicate the significance of differences from the expected values, i.e. fewer extinctions/lower degree of threat and more extinctions/higher degree of threat than expected, respectively. *Q* values equal zero when none of the simulated values crossed thresholds. Threatened species are those classified as such by BirdLife International (2011) in their IUCN Red List assessment

| Family | Number of extinct (and total) ultrataxa | Number of extinct (and total) species | Proportion extinct  ultrataxa | | Proportion extinct  species | | | Proportion  threatened species |
| --- | --- | --- | --- | --- | --- | --- | --- | --- |
| Acanthisittidae | 4 (7) | 1 (4) | | U 0.0000 | |  |  | |
| Acanthizidae | 1 (200) | 1 (65) | |  | |  |  | |
| Alaudidae | 0 (415) | 0 (91) | |  | |  |  | |
| Alcedinidae | 3 (316) | 0 (93) | |  | |  |  | |
| Alcidae | 1 (44) | 1 (24) | |  | |  |  | |
| Anatidae | 10 (244) | 6 (165) | | U 0.0065 | |  |  | |
| Ardeidae | 5 (142) | 4 (66) | |  | |  |  | |
| Bucerotidae | 1 (86) | 0 (55) | |  | |  |  | |
| Callaeatidae | 2 (5) | 1 (3) | | U 0.0226 | |  |  | |
| Campephagidae | 3 (304) | 0 (84) | |  | |  |  | |
| Caprimulgidae | 2 (223) | 1 (93) | |  | |  |  | |
| Cinclidae | 1 (30) | 0 (5) | |  | |  |  | |
| Cinclosomatidae | 1 (15) | 0 (5) | |  | |  |  | |
| Cisticolidae | 1 (459) | 0 (114) | |  | |  |  | |
| Columbidae | 21 (771) | 13 (318) | U 0.0028 | | U 0.0177 | | | U 0.0162 |
| Corvidae | 1 (360) | 1 (117) |  | |  | | |  |
| Cracidae | 1 (88) | 1 (51) |  | |  | | | U 0.0094 |
| Cracticidae | 1 (43) | 0 (12) |  | |  | | |  |
| Cuculidae | 4 (298) | 2 (143) |  | |  | | |  |
| Dasyornithidae | 1 (6) | 0 (3) |  | |  | | |  |
| Diomedeidae | 0 (23) | 0 (22) |  | |  | | | U 0.0000 |
| Dromaiidae | 3 (5) | 2 (3) | U 0.0000 | |  | | |  |
| Emberizidae | 7 (924) | 1 (308) |  | |  | | |  |
| Estrildidae | 1 (402) | 0 (136) |  | |  | | |  |
| Falconidae | 2 (154) | 2 (66) |  | |  | | |  |
| Formicariidae | 1 (174) | 0 (63) |  | |  | | |  |
| Fringillidae | 25 (463) | 14 (177) | U 0.0000 | | U 0.0000 | | |  |
| Gruidae | 0 (25) | 0 (15) |  | |  | | | U 0.0000 |
| Haematopodidae | 1 (17) | 1 (12) |  | |  | | |  |
| Hirundinidae | 1 (211) | 0 (82) |  | |  | | |  |
| Hydrobatidae | 1 (37) | 1 (23) | |  | |  |  | |
| Icteridae | 2 (320) | 1 (104) | |  | |  |  | |
| Maluridae | 3 (65) | 0 (27) | |  | |  |  | |
| Megapodidae | (0) 44 | 0 (21) | |  | |  | U 0.0112 | |
| Meliphagidae | 1 (402) | 1 (177) | |  | |  |  | |
| Mimidae | 1 (104) | 0 (34) | |  | |  |  | |
| Mohoidae | 5 (5) | 5 (5) | | U 0.0000 | | U 0.0000 | EX | |
| Monarchidae | 7 (269) | 5 (103) | |  | |  |  | |
| Muscicapidae | 1 (762) | 0 (286) | |  | |  |  | |
| Numididae | 1 (19) | 0 (6) | |  | |  |  | |
| Oriolidae | 4 (93) | 1 (3) | |  | |  |  | |
| Otididae | 1 (44) | 0 (25) | |  | |  |  | |
| Paridae | 1 (246) | 0 (51) | |  | |  |  | |
| Parulidae | 1 (371) | 1 (114) | |  | |  |  | |
| Petroicidae | 1 (135) | 0 (44) | |  | |  |  | |
| Phalacrocoracidae | 1 (59) | 1 (34) | |  | |  |  | |
| Phasianidae | 4 (559) | 1 (181) | |  | |  | U 0.0142 | |
| Picidae | 5 (720) | 1 (218) | |  | |  |  | |
| Podicipedidae | 3 (52) | 3 (22) | |  | |  |  | |
| Procellariidae | 3 (110) | 3 (82) | |  | |  | U 0.0000 | |
| Psittacidae | 29 (770) | 19 (374) | | U 0.0000 | | U 0.000 | U 0.0000 | |
| Pteroclididae | 1 (36) | 0 (16) | |  | |  |  | |
| Rallidae | 28 (325) | 21(156) | | U 0.0000 | | U 0.000 | U 0.0071 | |
| Raphidae | 2 (3) | 2 (2) | | U 0.0054 | | U 0.016 | EX | |
| Reguliidae | 1 (31) | 0 (6) | |  | |  |  | |
| Rhipiduridae | 2 (147) | 0 (42) | |  | |  |  | |
| Scolopacidae | 6 (149) | 3 (91) |  | |  | | |  |
| Sphenicidae | 0 (25) | 0 (18) |  | |  | | | U 0.0016 |
| Strigidae | 10 (560) | 3 (186) |  | |  | | |  |
| Struthionidae | 1 (5) | 0 (1) |  | |  | | |  |
| Sturnidae | 6 (233) | 4 (114) |  | |  | | |  |
| Sylviidae | 11 (677) | 2 (293) |  | |  | | |  |
| Thraupidae | 2 (539) | 0 (262) |  | |  | | |  |
| Threskiornithidae | 1 (48) | 1 (35) |  | |  | | |  |
| Timaliidae | 0 (931) | 0 (326) | L 0.000 | |  | | |  |
| Trochilidae | 5 (704) | 3 (338) |  | |  | | |  |
| Troglodytidae | 5 (450) | 0 (79) |  | |  | | |  |
| Turdidae | 10 (541) | 4 (174) |  | |  | | |  |
| Turnicidae | 1 (58) | 0 (16) |  | |  | | |  |
| Tyrannidae | 3 (1060) | 0 (415) |  | |  | | |  |
| Upupidae | 1 (8) | 1 (3) |  | |  | | |  |
| Zosteropidae | 5 (303) | 2 (100) |  | |  | | |  |

All families with extinctions are listed, plus those families where the numbers of extinct or threatened taxa are significantly differs from those expected. Extinct totals include taxa classified as ‘Extinct in the Wild’, ‘Possibly Extinct’ and ‘Possibly Extinct in the Wild’. Figures in parentheses give total number of taxa in a family (extant and extinct). Cells without a value had a non-significant *q*. Significant *p* values referred to in the text are not shown. EX means that there are no extant taxa in the family.
