# Supplementary material for: Global Patterns and Drivers of Avian Extinctions at the Species and Subspecies Level
Source: PLoS One. 2012 Oct 8;7(10):e47080. doi: 10.1371/journal.pone.0047080 (PMC3466226; doi:10.1371/journal.pone.0047080)
Supplement: References S1 — References for Tables S1, S2, S3 S4. (DOCX) [file pone.0047080.s005.docx]

**References**

Anon. (2000) Columnists’ Corner. Canberra Bird Notes 25: 120-125.

Anthony, A.W. (1901) The Guadalupe Wren. Condor 3: 73.

Ashby, E. (1927) The Grampian Range of Victoria and its Bird Life. Emu 26: 285–292.

Ashmole, N.P. (1963) Sub-fossil Bird Remains on Ascension Island. Ibis 103b: 382-389.

Bairlein, F., Alström, P., Aymí, R., Clement, P., Dyrcz, A., Gargallo, G., Hawkins, F., Madge, S., Pearson, D. and Svensson, L. (2006) Family Sylviidae (Old World Warblers). In Handbook of the Birds of the World Volume 11: Old World Flycatchers to Old World Warblers (J. del Hoyo, A. Elliott and D.A. Christie eds). Lynx Edicions, Barcelona.

Bangs, O. and Pernard, T.E. (1921) Notes on Some American Birds, Chiefly Neotropical. Bulletin of the Museum of Comparative Zoology 62: 23–93.

Baptista, L.F., Trail, P.W. and Horblit, H.M. (1997) Family Columbidae (Pigeons and Doves). In Handbook of the Birds of the World Volume 4: Sandgrouse to Cuckoos (J. del Hoyo, A. Elliott and J. Sargatal eds). Lynx Edicions, Barcelona.

Barrett, G., Silcocks, A., Barry, S., Cunningham, R. and Poulter, R. (2003).The New Atlas of Australian Birds. Birds Australia, Melbourne.

Barton, D.C., Lindquist, K.E., Henry, R.W. and Luna Mendoza, L.M. (2004) Landbird and Waterbird Notes from Isla Guadalupe, Mexico. Western Birds 35: 186–196.

Beintema, A.J. (1972) The History of the Island Hen (*Gallinula nesiotis*), The Extinct Flightless Gallinule of Tristan da Cunha. Bulletin of the British Ornithologists' Club 92: 106–113.

Bell, B.D. (1990). The Status and Management of the White-breasted White-eye and Other Birds on Norfolk Island. Royal Australasian Ornithologists Union, Melbourne.

Bibby, C.J. and Hill, D.A. (1987) Status of the Fuerteventura Stonechat *Saxicola dacotiae*. Ibis 129: 491–498.

BirdLife International (2001) Threatened Birds of Asia. BirdLife International, Cambridge.

BirdLife International (2011) BirdLife Data Zone. <http://www.birdlife.org/datazone/home>.

Black, A. (2011a) Western Australia, Home of the Grasswrens. Western Australian Journal of Ornithology 3: 1-12.

Black, A. (2011b) Subspecies of the Thick-billed Grasswren *Amytornis modestus* (Aves – Maluridae). Transactions of the Royal Society of South Australia 135: 26-38.

Blakers, M., Davies, S.J.J.F. and Reilly, P.N. (1984) The Atlas of Australian Birds. Melbourne University Press.

Blanvillain, C., Florent, C. and Thenot, V. (2002) Land Birds of Tuamotu Archipelago, Polynesia: Relative Abundance and Changes During the 20th Century with Particular Reference to the Critically Endangered Polynesian Ground-dove (*Gallicolumba erythroptera*). Biological Conservation 103: 139–149.

Boles, W.E. (2006) Family Rhipiduridae (Fantails). In Handbook of the Birds of the World Volume 11: Old World Flycatchers to Old World Warblers (J. del Hoyo, A. Elliott and D.A. Christie eds). Lynx Edicions, Barcelona.

Bond, J. (1956) Checklist of the Birds of the West Indies. Philadelphia Academy of Natural Sciences.

Bradley, P.E. (2000) The Birds of the Cayman Islands. British Ornithologists' Union, Peterborough.

Brazil, M.A. (1991) The Birds of Japan. Christopher Helm, London.

Brazil, M.A. (2009) Birds of East Asia: Eastern China, Taiwan, Korea, Japan and Eastern Russia. Christopher Helm, London.

Brewer, D. (2001) Wrens, Dippers and Thrashers. Christopher Helm, London.

Brooker, M.G., Ridpath, M.G., Estbergs, A.J., Bywater, J., Hart, D.S. and Jones, M.S. (1979) Bird Observations on the North-western Nullarbor Plain and Neighbouring Regions, 1967-1978. Emu 79: 176-190.

Brooks, T.M., Evans, T.D., Dutson, G.C.L., Anderson, G.Q.A., Asane, D.C., Timmins, R.J. and Toledo, A.G. (1992) The Conservation Status of the Birds of Negros, Philippines. Bird Conservation International 2: 273–302.

Buckley, P.A., Massiah, E.B., Hutt, M.B. and Buckley, F.G. (2009) The Birds of Barbados. British Ornithologists' Union, Peterborough.

Buden, D.W. (1993) Geographic Variation in the Scaly-breasted Thrasher *Margarops fuscus* with Descriptions of Three New Subspecies. Bulletin of the British Ornithologists' Club 113: 75–84.

Butchart, S. H. M., Crosby, M. J., Collar, N. J. and Tobias, J. A. (2005) Lost and poorly known birds: top targets for birders in Asia. Birding Asia. 3: 41–49.

Butynski, T. (2004) Uluguru Violet-backed Sunbirds *Anthreptes neglectus* at Tana, Kenya. Scopus 18: 62–64.

Byers, C., Olsson, U. and Curson, J. (1995) Buntings and Sparrows: A Guide to the Buntings and North American Sparrows. Pica Press, Robertsbridge.

Carboneras, C. (1992) Family Anatidae (Ducks, Geese and Swans). In Handbook of the Birds of the World Volume 1: Ostrich to Ducks (J. del Hoyo, A. Elliott and J. Sargatal eds). Lynx Edicions, Barcelona.

Carter, T. (1924) Birds of the Broome Hill District, Part 3. Emu 23: 306-318.

Cheke, A.S. (1987) An Ecological History of the Mascarene Islands, With Particular Reference to Extinctions and Introductions of Land Vertebrates. In Studies of Mascarene Island Birds (A.W. Diamond ed.). Cambridge University Press.

Cheke, A.S. (2006) Establishing Extinction Dates – The Curious Case of the Dodo *Raphus cucullatus* and the Red Hen *Aphanapteryx bonasia*. Ibis 148: 155-158.

Cibois, A., Thibault, J.-C.and Pasquet, E. (2008) Systematics of the Extinct Reed Warblers *Acrocephalus* of the Society Islands of Eastern Polynesia. Ibis 150: 365–376.

Clement, P. and Hathway, R. (2000) Thrushes.Christopher Helm, London.

Clement, P., Gregory, P.A. and Moeliker, C.W. (2006) Family Monarchidae (Monarch-flycatchers). In Handbook of the Birds of the World Volume 11: Old World Flycatchers to Old World Warblers (J. del Hoyo, A. Elliott and D.A. Christie eds). Lynx Edicions, Barcelona.

Clout, M.N. and Hay, J.R. (1981) South Island Kokako (*Callaeas cinerea cinerea*) in Nothofagus Forest. Notornis 28: 256–259.

Collar, N.J. (1996) Family Otididae (Bustards). In Handbook of the Birds of the World Volume 3: Hoatzin to Auks (J. del Hoyo, A. Elliott and J. Sargatal eds). Lynx Edicions, Barcelona.

Collar, N.J. (1997) Family Psittacidae (Parrots). In Handbook of the Birds of the World Volume 4: Sandgrouse to Cuckoos (J. del Hoyo, A. Elliott and J. Sargatal eds). Lynx Edicions, Barcelona.

Collar, N.J. (2005) Family Turdidae (Thrushes). In Handbook of the Birds of the World Volume 10: Cuckoo-shrikes to Thrushes (J. del Hoyo, A. Elliott and D.A. Christie eds). Lynx Edicions, Barcelona.

Collar, N.J. and Wege, D.C. (1995) The Distribution and Conservation Status of the Bearded Tachuri *Polystictus pectoralis*. Bird Conservation International 5: 367–390.

Collar, N.J., Crosby, M.J. and Stattersfield, A.J. (1994) Birds to Watch 2: The World List of Threatened Birds. BirdLife International, Cambridge.

Collar, N.J., Newton, I., Clement, P. and Arkhipov, V. (2010) Family Fringillidae (Finches). In Handbook of the Birds of the World Volume 15: Weavers to New World Warblers (J. del Hoyo, A. Elliott and D.A. Christie eds). Lynx Edicions, Barcelona.

Collen, B., Purvis, A. and Mace, G.M. (2010) When is a Species Really Extinct? Testing Extinction Inference from a Sighting Record to Inform Conservation Assessment. Diversity and Distributions 16: 755-764.

Cooper, J.H. and Tennyson, A.J.D. (2004) New Evidence on the Life and Death of Hawkins' Rail (*Diaphorapteryx hawkinsi*): Moriori Accounts Recorded by Sigvard Dannefaerd and Alexander Shand. Notornis 51: 212–216.

Curio, E. (1994) Ornithological Observations During a (Preliminary) Philippines Conservation Expedition in 1993. Okologie der Vögel 16: 613–623.

Danforth, S.T. (1934) The Birds of Antigua. Auk 51: 350–364.

Davison, G.W.H. and McGowan, P.J.K. (2009) Is the Double-banded Argus *Argusianus bipunctatus* a Valid Species? Birding Asia 12: 94-96.

De Juana, E. (1994) Family Tetraonidae (Grouse). In Handbook of the Birds of the World Volume 2: New World Vultures to Guineafowl (J. del Hoyo, A. Elliott and J. Sargatal eds). Lynx Edicions, Barcelona.

De Juana, E. (1997) Family Pteroclididae (Sandgrouse). In Handbook of the Birds of the World Volume 4: Sandgrouse to Cuckoos (J. del Hoyo, A. Elliott and J. Sargatal eds). Lynx Edicions, Barcelona.

De Zoysa, N.D. and Raheem, R. (1987) Sinharaja: A Rainforest in Sri Lanka. March for Conservation, Colombo.

del Hoyo, J. (1994) Family Cracidae (Chachalacas, Guans and Curassows). In Handbook of the Birds of the World Volume 2: New World Vultures to Guineafowl (J. del Hoyo, A. Elliott and J. Sargatal eds). Lynx Edicions, Barcelona.

Desjardins, J. (1832) Troisième Rapport Annual Travaille Société d'Histoire Naturelle Ile Maurice.Unpublished report.

Dickerman, R.W. (1965) The Juvenile Plumage and Distribution of *Cassidix palustris* (Swainson). Auk 82: 268–270.

Dickinson, E.C. ed. (2003) The Howard and Moore Complete Checklist of the Birds of the World. Christopher Helm, London.

Dickison, D. (1926) When Did the Tasmanian Emu Become Extinct? Emu 25: 213.

Disney, H.J. de S. and Smithers, C.N. (1972).The Distribution of Terrestrial and Freshwater Birds on Lord Howe Island, In Comparison with Norfolk Island. Australian Zoologist 17: 1–11.

Dod, J.S. (1992) Endangered & Endemic Birds of the Dominican Republic. Cypress House Press, Fort Bragg.

Donaghho, W.R. (1963) A Resume of the 'Extinct' Hawaiian Species, With Listings of Sight Records and Reports During My Sojourn in the Islands. Elepaio 23: 55–58.

Donegan, T.M. (2004) Aves de la Sabana de Bogotá, Guía de Campo (Birds of the Sabana de Bogotá, Field Guide). Wilson Bulletin 116: 113–114.

Donegan, T.M., Huertas, B.C., Briceño, E.L., Arias, J.J. and González, C.E. (2003a) Search for the Magdalena Tinamou: Project Report. Unpublished report.

Donegan, T.M., Huertas, B.C. and Briceño, E.L. (2003b) Status of the Magdalena Tinamou *Crypturellus saltuarius* in the Type Locality and Surrounding Lower Magdalena Valley. Cotinga 19: 34–39.

Dove, H.S. (1924) Notes on the Tasmanian Emu. Emu 23: 221–222.

Dutson, G. (2011) Birds of Melanesia: The Bismarcks, Solomons, Vanuatu and New Caledonia. Princeton University Press.

Ekstrom, J.M.M., Jones, J.P.G., Willis, J., Tobias, J., Dutson, G. and Barré, N. (2002) New Information on the Distribution, Status and Conservation of Terrestrial Bird Species in Grande Terre, New Caledonia. Emu 102: 197–207.

Etheridge, R. (1889) The General Zoology of Lord Howe Island. Australian Museum Memoirs 2: 3-42.

Evans, T.D., Dutson, G.C.L. and Brooks, T.M. (1993) Cambridge Philippines Rainforrest Project 1991, Final Report. BirdLife International, Cambridge.

Fitzpatrick, J., Bates, J., Bostwick, K., Caballero, I., Clock, B., Farnsworth, A., Hosner, P., Joseph, L., Langham, G., Lebbin, D., Mobley, J., Robbins, M., Scholes, E., Tello, J., Walther, B. and Zimmer, K. (2004) Family Tyrannidae (Tyrant-flycatchers). In Handbook of the Birds of the World Volume 9: Cotingas to Pipits and Wagtails (J. del Hoyo, A. Elliott and D.A. Christie eds). Lynx Edicions, Barcelona.

Fjeldså, J. (1993) The Decline and Probable Extinction of the Colombian Grebe *Podiceps andinus*. Bird Conservation International 3: 221–234.

Fleming, C.A. (1939) Birds of the Chatham Islands. Emu 38: 380–413.

Flint, P.R. and Stewart, P.F. (1983) The Birds of Cyprus. British Ornithologists' Union, Peterborough.

Folch, A. (1992) Family Struthionidae (Ostrich). In Handbook of the Birds of the World Volume 1: Ostrich to Ducks (J. del Hoyo, A. Elliott and J. Sargatal eds). Lynx Edicions, Barcelona.

Forshaw, J.M. (1989) Parrots of the World. Macmillan, London.

Freile, J.F., Parra, J.L. and Graham, C.H. (2010) Distribution and Conservation of *Grallaria* and *Grallaricula* (Grallariidae) Antpittas in Ecuador. Bird Conservation International 20: 410–431.

Fry, C.H. (2001) Family Coraciidae (Rollers). In Handbook of the Birds of the World Volume 6: Mousebirds to Hornbills (J. del Hoyo, A. Elliott and J. Sargatal eds). Lynx Edicions, Barcelona.

Fryer, J.C.F. (1911) The Structure and Formation of Aldabra and Neighbouring Islands - With Notes on Their Flora and Fauna. Transactions of the Linnaean Society 14: 397–442.

Fuller, E. (2000) Extinct Birds.Oxford University Press.

Garnett, S.T., Szabo, J.K. and Dutson, G.C.L. (2011) The Action Plan for Australian Birds 2010. CSIRO Publishing, Collingwood.

Garnett, S.T., Olsen, P., Butchart, S.H.M. and Hoffman, A.A. (2011) Did Hybridization Save the Norfolk Island Boobook Owl *Ninox novaeseelandiae undulata*? Oryx 45: 500–504.

Gibbs, D., Barnes, E. and Cox, J. (2001) Pigeons and Doves: A Guide to the Pigeons and Doves of the World. Christopher Helm, London.

Glauert, L. (1944) Bristle-birds in Western Australia. Emu 44: 334.

Gorresen, P.M., Camp, R.J., Reynolds, M.H., Woodworth, B.L. and Pratt, T.K. (2009) Status and Trends of Native Hawaiian Songbirds. In Conservation Biology of Hawaiian Forest Birds(T.K. Pratt, C.T. Atkinson, P.C. Banko, J.D. Jacobi and B.L. Woodworth eds). Yale University Press.

Gosler, A. and Clement, P. (2007) Family Paridae (Tits and Chickadees). In Handbook of the Birds of the World Volume 12: Picathartes to Tits and Chickadees (J. del Hoyo, A. Elliott and D.A. Christie eds). Lynx Edicions, Barcelona.

Grantham, M.J. (2000) Birds of Alas Purwo National Park, East Java. Kukila 11: 97–121.

Graves, G.R. and Olson, S.L. (1987) *Chlorostilbon bracei* Lawrence, An Extinct Species of Hummingbird from New Providence Island, Bahamas. Auk 104: 296-302.

Greenway, J.C. (1967) Extinct and Vanishing Birds of the World. Dover Publications, New York.

Gregory, P. (2007) Family Dasyornithidae (Bristlebirds). In Handbook of the Birds of the World Volume 12: Picathartes to Tits and Chickadees (J. del Hoyo, A. Elliott and D.A. Christie eds). Lynx Edicions, Barcelona.

Grinnell, J. (1928) A Distributional Summation of the Ornithology of Lower California. University of California Publications in Zoology 32: 1–300.

Hamel, P.B. (2011) Bachman's Warbler (*Vermivora bachmanii*). In The Birds of North America Online (A. Poole ed.). Cornell Laboratory of Ornithology, Ithaca.

Hamilton, A. (1894) Notes on a Visit to Macquarie Island. Transactions and Proceedings of the New Zealand Institute 27: 559–579.

Haynes-Sutton, A., Downer, A. and Sutton, R. (2009) A Photographic Guide to the Birds of Jamaica. Christopher Helm, London.

Hermes, N. (1985) Birds of Norfolk Island.Wonderland Publications, Norfolk Island.

Hindwood, K.A. (1940) The Birds of Lord Howe Island. Emu 40: 1–86

Holmes, G. (1996) Distribution and Status of the Southern Star Finch. Sunbird 26: 49–59.

Holmes, G. (1998) A Review of the Distribution, Status and Ecology of the Star Finch *Neochmia ruficauda* in Queensland. Australian Bird Watcher 17: 278–289.

Holyoak, D.T. (1973) An Undescribed Extinct Parrot from Mauritius. Ibis 115: 417–418.

Holyoak, D.T. and Thibault, J.-C.(1977) *Halcyon gambieri gambieri* Oustalet, An Extinct Kingfisher from Mangareva, South Pacific Ocean. Bulletin of the British Ornithologists' Club 97: 21–23.

Holyoak, D.T. and Thibault, J.-C.(1984) Contribution à l'Etude des Oiseaux de Polynésie Orientale. Memoirs du Museum d'Histoire Naturelle A 127: 1–209.

Houston, W., Porter, G., O'Neill, P. and Elder, R. (2004) The Ecology of the Critically Endangered Yellow Chat *Epthianura crocea macgregori* on Curtis Island. Sunbird 34: 10–23.

Hull, A.F.B. (1909) The Birds of Lord Howe and Norfolk Islands. Proceedings of the Linnaean Society of New South Wales 34: 636-693.

Hume, J.P. and Walters, M. (2012) Extinct Birds. T and AD Poyser, London.

Jehl, J.R. and Parkes, K.C. (1982) The Status of the Avifauna of the Revillagigedo Islands, Mexico. Wilson Bulletin 94: 1–19.

Johansson, U.S., Pasquet, E. and Irestedt, M. (2011) The New Zealand Thrush: An Extinct Oriole. PLoS One 6: e24317.

Johnsgard, P.A. (1991) Bustards, Hemipodes, And Sandgrouse: Birds of Dry Places. Oxford University Press.

Johnston, D.W. (1969) The Thrushes of Grand Cayman Island, BWI. Condor 71: 120–128.

Joines, S. (1985) America's Extinct Parrot. ZooNooz 58: 4–9.

Jones, P. and Tye, A. (2006) The Birds of São Tomé & Príncipe with Annobón. British Ornithologists' Union, Peterborough.

Joseph, L. and Stockwell, D. (2002) Climatic Modeling of the Distribution of Some *Pyrrhura* Parakeets of Northwestern South America with Notes on Their Systematics and Special Reference to *Pyrrhura caeruleiceps* Todd, 1947. Ornitologia Neotropical 13: 1–8.

Kaeding, H.B. (1905) Birds from the West Coast of Lower California and Adjacent Islands. Condor 7: 134–138.

Keith, A.R., Wiley, J.W., Latta, S.C. and Ottenwalder, J.A. (2003) The Birds of Hispaniola: Haiti and the Dominican Republic. British Ornithologists' Union, Peterborough.

Kemp, A.C. (2001) Family Bucerotidae (Hornbills). In Handbook of the Birds of the World Volume 6: Mousebirds to Hornbills (J. del Hoyo, A. Elliott and J. Sargatal eds). Lynx Edicions, Barcelona.

Kennerley, P. and Pearson, D. (2010) Reed and Bush Warblers.Christopher Helm, London.

Knox, A.G. and Walters, M.P. (1994) Extinct and Endangered Birds in the Collections of the Natural History Museum. British Ornithologists' Club, Tring.

Kroodsma, D. and Brewer, D. (2005) Family Troglodytidae (Wrens). In Handbook of the Birds of the World Volume 10: Cuckoo-shrikes to Thrushes (J. del Hoyo, A. Elliott and D.A. Christie eds). Lynx Edicions, Barcelona.

Latta, S., Wiley, J.W., Rimmer, C., Raffaele, H.A., Keith, A.R., McFarland, K. and Fernandez, E. (2006) Birds of the Dominican Republic and Haiti.Christopher Helm, London.

Le Souëf, D. (1904) The Extinct Tasmanian Emu. Emu 3: 229–231.

Leck, C.F. (1975) Notes on Unusual and Rare Birds of St Croix. Condor 77: 107.

Levesque, A. and Mathurin, A. (2008) Guadeloupe. In Important Bird Areas in the Caribbean (D.C. Wege and A. Anadon-Irizarry eds). BirdLife International, Cambridge.

Lindsey, G.D., Van der Werf, E.A., Baker, H. and Baker, P. (1998) Hawai'i (*Hemignathus virens*), Kaua'i (*Hemignathus kauaiensis*), O'ahu (*Hemignathus chloris*) and Greater 'Amakihi (*Hemignathus sagittirostris*). In The Birds of North America Online (A. Poole ed.). Cornell Laboratory of Ornithology.

Liukkonen-Anttila, T., Uimaniemi, L., Orell, M. and Lumme, J. (2002) Mitochondrial DNA Variation and the Phylogeography of the Grey Partridge (*Perdix perdix*) in Europe: From Pleistocene History to Present Day Populations. Journal of Evolutionary Biology 15: 971–982.

Lovari, S. (1975) A Partridge in Danger. Oryx 13: 203–204.

Mackenzie, D. (1968) The Birds and Seals of the Bishop and Clerk Islets, Macquarie Island. Emu 67: 241–245.

Manabu, K., Tooru, M. and Fumio, S. (2002) Two Forms of Bush Warbler *Cettia diphone* Occur on Okinawajima Island: Re-evaluation of *C. d. riukiuensis* and *C. d. restricta* by Multivariate Analyses. Journal of the Yamashina Institute for Ornithology 33: 148-167.

Marchant, S. and Higgins, P.J. (1993) Handbook of Australian, New Zealand and Antarctic Birds Volume 2: Raptors to Lapwings.Oxford University Press.

Marks, J.S., Cannings, R.J. and Mikkola, H. (1999) Family Strigidae (Typical Owls). In Handbook of the Birds of the World Volume 5: Barn-owls to Hummingbirds (J. del Hoyo, A. Elliott and J. Sargatal eds). Lynx Edicions, Barcelona.

Martínez, I. (1994) Family Numididae (Guineafowl). In Handbook of the Birds of the World Volume 2: New World Vultures to Guineafowl (J. del Hoyo, A. Elliott and J. Sargatal eds). Lynx Edicions, Barcelona.

Martínez-Vilalta, A. and Motis, A. (1992) Family Ardeidae (Herons). In Handbook of the Birds of the World Volume 1: Ostrich to Ducks (J. del Hoyo, A. Elliott and J. Sargatal eds). Lynx Edicions, Barcelona.

Mason, I.J. and Schodde, R. (1997) Bird Survey of the Tiwi Islands, October 1996. CSIRO Publishing, Collingwood.

Masters, J.R. and Milhinch, A.L. (1974) Birds of the Shire of Northam, About 100 km East of Perth. Emu 74: 228-244.

Matheu, E. and del Hoyo, J. (1992) Family Threskiornithidae (Ibises and Spoonbills). In Handbook of the Birds of the World Volume 1: Ostrich to Ducks (J. del Hoyo, A. Elliott and J. Sargatal eds). Lynx Edicions, Barcelona.

Matteucci, C. (1988) The Grey Partridge in Italy: History, Present Status, Distribution and Perspectives in Management. In Proceedings of the International Symposium Common Partridge (*Perdix perdix*) (Polish Hunting Association ed.). Polish Hunting Association.

McAllan, I.A.W. (1987) Early Records of the Thick-billed Grasswren *Amytornis textilis* and Striated Grasswren *Amytornis striatus* in New South Wales. Australian Birds 28: 65-70.

McAllan, I.A.W., Curtis, B.R., Hutton, I. and Cooper, R.M. (2004) The Birds of the Lord Howe Island Group: A Review of Records. Australian Field Ornithology 21: 1–82.

McGregor, R.C. (1905) Further Notes on Birds from Ticao, Cuyo, Culion, Calayan, Lubang, And Luzon. Bulletin of the Bureau of Government Laboratories of Manila 25: 25–34.

McKean, J.L., Evans, O., Lewis, J.H. (1976) Notes on the Birds of Norfolk Island. Notornis 23: 299–301.

McKinley, D. (1985) The Carolina Parakeet in Florida. Florida Ornithological Society.

Meinertzhagen, R. (1925) May in Madeira. Ibis 67: 600–621.

Merrill, G.W. (1967) Plains Sharp-tailed Grouse. In New Mexico Wildlife Management (W.S. Huey ed.). New Mexico Department of Game and Fish.

Milligan, A.W. (1901) Description of a New Bristlebird (Sphenura). Emu 1: 67–69.

Mlíkovský, J. (2004) Extinction of the Dodo *Raphus cucullatus* (Aves: Raphidae): Dating Reconsidered. Journal of the National Museum, Natural History Series 173: 111-112.

Moore, J.L. (1985) Ensign Best's Bird Observations on Norfolk Island. Notornis 32: 319–322.

Moreno, J.A. (1998) Status of the Virgin Islands Screech-owl. Journal of Field Ornithology 69: 557–562.

Morgan, A.M. and Sutton, J. (1928) A Critical Description of Some Recently Discovered Bones of the Extinct Kangaroo Island Emu (*Dromaius diemenianus*). Emu 28: 1–19.

Morris, P. and Hawkins, F. (1998) Birds of Madagascar: A Photographic Guide. Pica Press, Robertsbridge.

Munro, G.C. (1960) Birds of Hawaii.Bridgeway Press Books, Austin.

Nash, S.V. (1993) Concern About Trade in Red-and-blue Lories. Traffic Bulletin 13: 93–96.

Nellis, D.W. (1979) Record of Puerto Rican Screech Owl, Turkey Vulture and Osprey from St Croix, US Virgin Islands. Wilson Bulletin 91: 148–149.

Nicoll, M.J. (1906) On the Birds Collected and Observed During the Voyage of the Valhalla, RYS, From November 1905 to May 1906. Ibis 48: 666–712.

Norton, R.L. (1986) The Spring Migration: West Indies Region. American Birds 40: 528–529.

Noske, R., Prawiradilaga, D.M., Drynan, D., Leishman, A. and Rutherford, W. (2011) Understorey Birds of Cikaniki Research Station, Gunung Halimun-Salak National Park, West Java: Report of the Indonesian Bird Banding Scheme Training Programme. Kukila 15: 50–65.

Olsen, P. (2007) Glimpses of Paradise: The Quest for the Beautiful Parakeet. National Library of Australia, Canberra.

Olson, S.L. and James, H.F. (1995) Nomenclature of the Hawaiian Akialoas and Nukupuus (Aves: Drepanidini). Proceedings of the Biological Society of Washington 108: 373–387.

Otto, R. (2003) Threatened Wildlife and Old-growth Forest Survey of the Arroyo Durango, Sierra Madre Occidental, Mexico.Unpublished report.

Paguntalan, L.M.J. and Jakosalem, P.G. (2008) Significant Records of Birds in Forests on Cebu Island, Central Philippines. Forktail 24: 48–56.

Parker, D.G., Egan, D. and Ballestrin, M.L. (2010) Recent Observations of the Thick-billed Grasswren *Amytornis textilis modestus* in New South Wales. Australian Field Ornithology 27: 159-164.

Parker, S.A. (1972) Remarks on Distribution and Taxonomy of the Grass Wrens *Amytornistextilis*, *modestus* and *purnelli*. Emu 72: 157–166.

Paton, D.C., Carpenter, G. and Sinclair, R.G. (1994) A Second Bird Atlas in the Adelaide Region. Part 1: Changes in the Distributions of Birds 1974–75 Versus 1984–85. South Australian Ornithologist 31: 151–193.

Penny, M. (1974) The Birds of Seychelles and the Outlying Islands. HarperCollins, London.

Pfennigwerth, S. (2010) ‘The Mighty Cassowary’: The Discovery and Demise of the King Island Emu. Archives of Natural History 37: 74–90.

Phillipps, W.J. (1959) The Last (?) Occurrence of Notornis in the North Island. Notornis 8: 93–94.

Piersma, T., Van Gils, J. and Wiersma, P. (1996) Family Scolopacidae (Sandpipers and Allies). In Handbook of the Birds of the World Volume 3: Hoatzin to Auks (J. del Hoyo, A. Elliott and J. Sargatal eds). Lynx Edicions, Barcelona.

Pratt, H.D. (1994) Avifaunal Change in the Hawaiian Islands, 1893–1993. Studies in Avian Biology 15: 103–118.

Pratt, H.D. (2005) The Hawaiian Honeycreepers. Oxford University Press.

Pratt, H.D. (2010) Family Drepanididae (Hawaiian Honeycreepers). In Handbook of the Birds of the World Volume 15: Weavers to New World Warblers (J. del Hoyo, A. Elliott and D.A. Christie eds). Lynx Edicions, Barcelona.

Pratt, H.D., Bruner, P.L. and Berrett, D.G. (1987) A Field Guide to the Birds of Hawaii and the Tropical Pacific. Princeton University Press.

Pratt, T.K. and Pyle, R.L. (2000) Nukupu'u in the Twentieth Century: Endangered Species or Phantom Presence? Elepaio 60: 35–41.

Rabor, D.S. (1959) The Impact of Deforestation on Birds of Cebu, Philippines, With New Records for That Island. Auk 76: 37–43.

Raffaele, H.A., Wiley, J.W., Garrido, O., Keith, A. and Raffaele, J. (1998) Birds of the West Indies. Christopher Helm, London.

Recher, H.F. and Clark, S.S. (1974) A Biological Survey of Lord Howe Island with Recommendations for the Conservation of the Island's Wildlife. Biological Conservation 6: 263–273.

Reichel, J.D., Wiles, G.J. and Glass, P.O. (1992) Island Extinctions: The Case of the Endangered Nightingale Reed-warbler. Wilson Bulletin 104: 44–54.

Remsen, J.V. (2003) Family Furnariidae (Ovenbirds). In Handbook of the Birds of the World Volume 8: Broadbills to Tapaculos (J. del Hoyo, A. Elliott and D.A. Christie eds). Lynx Edicions, Barcelona.

Reynolds, M.H. and Snetsinger, T.J. (2001) The Hawaii Rare Bird Search 1994–1996. Studies in Avian Biology 22: 133–143.

Ridgway, R. (1895) On Birds Collected by Dr W.L. Abbott in the Seychelles, Amirantes, Gloriosa, Assumption, Aldabra and Associated Islands, With Notes on Habits Etc., By the Collector. Proceedings of the United States National Museum 18: 509–546.

Riley, J. (1997) Biological Surveys and Conservation Priorities on the Sangihe and Talaud Islands, Indonesia.CSB Conservation Publications, Cambridge.

Riley, J. (2002) Population Sizes and the Status of Endemic and Restricted-range Bird Species on Sangihe Island, Indonesia. Bird Conservation International 12: 53–78.

Ripley, S.D. and Rabor, D.S. (1956) Birds from Canlaon Volcano in the Highlands of Negros Island in the Philippines. Condor 58: 283–291.

Robinson, D. (1988). Ecology and Management of the Scarlet Robin, White-breasted White-eye and Long-billed White-eye on Norfolk Island.Australian National Parks and Wildlife Service, Canberra.

Robson, C. (2007) Family Paradoxornithidae (Parrotbills). In Handbook of the Birds of the World Volume 12: Picathartes to Tits and Chickadees (J. del Hoyo, A. Elliott and D.A. Christie eds). Lynx Edicions, Barcelona.

Rothschild, W. (1900) The Avifauna of Laysan and the Neighbouring Islands with a Complete History to Date of the Birds of the Hawaiian Possession. R.H. Porter, London.

Ryan, P.G., Dean, W.R.J., Madge, S.C. and Pearson, D.J. (2006) Family Cisticolidae (Cisticolas and Allies). In Handbook of the Birds of the World Volume 11: Old World Flycatchers to Old World Warblers (J. del Hoyo, A. Elliott and D.A. Christie eds). Lynx Edicions, Barcelona.

Sabo, S.R. (1982) The Rediscovery of Bishop's Oo on Maui. Elepaio 42: 69–70.

Salaman, P., Donegan, T.M. and Prŷs-Jones, R. (2009) A New Subspecies of Brown-banded Antpitta *Grallaria milleri* from Antioquia, Colombia. Bulletin of the British Ornithologists Club 129: 5-17.

Schodde, R. and Mason, I.J. eds (1997) Zoological Catalogue of Australia 37.2: Aves (Columbidae to Coraciidae). CSIRO Publishing, Melbourne.

Schodde, R., Fullagar, P. and Hermes, N. (1983) A Review of Norfolk Island Birds: Past and Present.Australian National Parks and Wildlife Service, Canberra.

Schuchmann, K.L. (1999) Family Trochilidae (Hummingbirds). In Handbook of the Birds of the World Volume 5: Barn-owls to Hummingbirds (J. del Hoyo, A. Elliott and J. Sargatal eds). Lynx Edicions, Barcelona.

Scott, J.H. (1882) Macquarie Island. Transactions and Proceedings of the New Zealand Institute 15: 484–493.

Scott, J.M., Mountainspring, S., Ramsey, F.L. and Kepler, C.B. (1986) Forest Bird Communties of the Hawiian Islands: Their Dynamics, Ecology, And Conservation. Cooper Ornithological Society, California.

Seitre, R. and Seitre, J. (1991) Causes de Disparition des Oiseaux Terrestres de Polynésie Française. South Pacific Regional Environment Programme, Nouméa.

Seitre, R. and Seitre, J. (1992) Causes of Land-bird Extinctions in French Polynesia. Oryx 26: 215–222.

Serventy, D.L. and Whittell, H.M (1967) Birds of Western Australia. Lamb Publishers, Perth.

Sharland, M.S.R. (1929) Land birds of Lord Howe Island. Emu 29: 5–11.

Skerrett, A. and Disley, T. (2011) Birds of Seychelles.Christopher Helm, London.

Smithers, C.N. and Disney, H.J. de S. (1969) The Distribution of Terrestrial and Freshwater Birds on Norfolk Island. Australian Zoologist 15: 127–140.

Stattersfield, A.J., Crosby, M.J., Long, A.J. and Wege, D.C. (1998) Endemic Bird Areas of the World: Priorities for Biodiversity Conservation. BirdLife International, Cambridge.

Stiles, F.G., Roselli, L. and Bohórquez, C.I. (1999) New and Noteworthy Records of Birds from the Middle Magdalena Valley of Colombia. Bulletin of the British Ornithologists' Club 119: 113–129.

Streets, T.H. (1877) Contributions to the Natural History of the Hawaiian and Fanning Islands and Lower California, Made in Connection with the US North Pacific Surveying Expedition, 1873–1875. Bulletin of the United States National Museum 7: 3–172.

Taylor, P.B. (1996) Family Rallidae (Rails, Gallinules and Coots). In Handbook of the Birds of the World Volume 3: Hoatzin to Auks (J. del Hoyo, A. Elliott and J. Sargatal eds). Lynx Edicions, Barcelona.

Taylor, P.B. and Van Perlo, B. (1998) Rails: A Guide to the Rails, Crakes, Gallinules and Coots of the World. Pica Press, Robertsbridge.

Taylor, R.H. (1979) How the Macquarie Island Parakeet Became Extinct. New Zealand Journal of Ecology 2: 42–45.

Tennyson, A.J.D. and Martinson, P. (2006) Extinct Birds of New Zealand. Te Papa Press, Wellington.

Tenorio, J.C. et al. (1979) Ornithological Survey of Wetlands in Guam, Saipan, Tinian and Pagan. US Army Corps of Engineers, Honolulu.

Terauds, A., Gales, R., Baker, G.B. and Alderman, R. (2006) Foraging Areas of Black-browed and Grey-headed Albatrosses Breeding on Macquarie Island in Relation to Marine Protected Areas. Aquatic Conservation 16: 133–146.

Thayer, J.E. and Bangs, O. (1908) The Present State of the Ornis of Guadaloupe Island. Condor 10: 101-106.

Thibault, J.-C.(1973) Notes Ornithologiques Polynesiennes. Alauda 38: 255–273.

Thibault, J.-C.and Meyer, J.-Y. (2001) Contemporary Extinction and Population Declines of the Monarchs (*Pomarea* spp.) in French Polynesia, South Pacific. Oryx 35: 73–80.

Tobias, J. A., Butchart, S. H. M. and Collar, N. J. (2006) Lost and found: a gap analysis for the Neotropical avifauna. Neotropical Birding 1: 4–22.

Turbott, E.G. (1990) Checklist of the Birds of New Zealand. Random Century, Auckland.

Underwood, C.F. (1896) A List of Birds Collected or Observed on the Lower, Southern, And South-western Slopes of the Volcano of Miravalles and on the Lower Lands Extending to Bagaces in Costa Rica, With a Few Observations on Their Habits. Ibis 38: 431–451.

Van Balen, B. (2008) Family Zosteropidae (White-eyes). In Handbook of the Birds of the World Volume 13: Penduline-tits to Shrikes (J. del Hoyo, A. Elliott and D.A. Christie eds). Lynx Edicions, Barcelona.

Vargas, H. and Bensted-Smith, R. (2000) Past and Present Ornithology in Galápagos. In Proceedings of the Symposium Science and Conservation in Galápagos (N. Sitwell, L. Baert and G. Cuppois eds). Royal Belgian Institute of Natural Sciences, Brussels.

Vestjens, W.J.M. (1963). Remains of the Extinct Banded Rail at Macquarie Island. Emu 62: 249–250.

Voisin, C. and Voisin, J.-F.(1995) A Fifth Specimen of the Tahiti Parakeet. Bulletin of the British Ornithologists' Club 115: 262-263.

Walters, M. (1993) On the Status of the Christmas Island Sandpiper *Aechmorhynchuscancellatus*. Bulletin of the British Ornithologists' Club 113: 97–102.

Walters, M.J. (1992) A Shadow and a Song. Chelsea Green, White River Junction.

Walther, B. and Jones, P. (2008) Family Oriolidae (Orioles). In Handbook of the Birds of the World Volume 13: Penduline-tits to Shrikes (J. del Hoyo, A. Elliott and D.A. Christie eds). Lynx Edicions, Barcelona.

Warner, D.W. (1947) The Ornithology of New Caledonia and the Loyalty Islands. PhD Thesis, Cornell University.

Watling, D. (2004) A Guide to the Birds of Fiji & Western Polynesia, Including American Samoa, Niue, Samoa, Tokelau, Tonga, Tuvalu and Wallis & Futuna. Environmental Consultants, Suva.

Wehtje, W., Walter, H.S., Rodríguez-Estrella, R., Llinas, J. and Castellanos-Vera, A. (1993) An Annotated Checklist of the Birds of Isla Socorro, Mexico. Western Birds 24: 1–16.

Wetmore, A. (1927) The Birds of Porto Rico and the Virgin Islands. New York Academy of Sciences.

Wetmore, A., Pasquier, R.F. and Olson, S.L. (1984) The Birds of the Republic of Panamá Part 4, Passeriformes: Hirundinidae (Swallows) to Fringillidae (Finches). Smithsonian Institution Press, Washington.

Whitley, G.P. (1971). Field Notes on Birds by Thomas Carter. Western Australian Naturalist 12: 41–44.

Whittell, H.M. (1933) The Birds of Bridgetown District, South-west Australia. Emu 32: 182–189.

Whittell, H.M. (1936) The Bristle-birds of Western Australia. Emu 35: 197-201.

Wiedenfeld, D.A. (2006) Aves, The Galapagos Islands, Ecuador. Check List 2: 1–27.

Wilbur, S.R. (1987) Birds of Baja California.University of California Press.

Williams, G.R. and Harrison, M. (1972) The Laughing Owl *Sceloglaux albifacies* (Grey 1844), A General Survey of a Near-extinct Species. Notornis 19: 4–19.

Winkler, H. and Christie, D.A. (2001) Family Picidae (Woodpeckers). In Handbook of the Birds of the World Volume 7: Jacamars to Woodpeckers (J. del Hoyo, A. Elliott and J. Sargatal eds). Lynx Edicions, Barcelona.

Woinarski, J., Brennan, K., Hempel, C., Armstrong, M., Milne, D., and Chatto, R. (2003) Biodiversity Conservation on the Tiwi Islands, Northern Territory, Part 2: Fauna.Department of Infrastructure Planning and Environment, Darwin.

Woodall, P.F. (2001) Family Alcedinidae (Kingfishers). In Handbook of the Birds of the World Volume 6: Mousebirds to Hornbills (J. del Hoyo, A. Elliott and J. Sargatal eds). Lynx Edicions, Barcelona.
